# Interaction–structure coupling enables high-flux enantioselective transport in lamellar membranes

**DOI:** 10.1039/d6sc03058k

**Published:** 2026-06-19

**Authors:** Siqi Han, Furong Yuan, Honghong Cao, Wentao Han, Ximeng Chen, Hongyan Wan, Zhan Li

**Affiliations:** a MOE Frontiers Science Center for Rare Isotopes, Lanzhou University Lanzhou 730000 China hywan@lzu.edu.cn liz@lzu.edu.cn; b School of Accounting, Lanzhou University of Finance and Economics Lanzhou 730020 China; c School of Nuclear Science and Technology, Lanzhou University Lanzhou 730000 China; d School of Chemistry and Chemical Engineering, Qinghai Nationalities University Xining 810007 China

## Abstract

Enantioselective transport in solid membranes is often limited because weak stereospecific interactions are difficult to translate into substantial kinetic discrimination under fast diffusion. Here we show that such weak recognition can be amplified through an interaction–structure coupling mechanism in lamellar membranes. Helical ssDNA-wrapped single-walled carbon nanotubes were intercalated into graphene oxide laminates to construct continuous chiral transport pathways with adaptive confined interfaces. Opposite phenylalanine enantiomers induce distinct reconfiguration of the lamellar galleries, leading to differentiated interlayer spacings and a pronounced split in apparent migration barriers. After reduction, the resulting membrane exhibits a large activation-energy difference between l- and d-phenylalanine transport, enabling high-flux enantioselective permeation, separation factors up to 12.59, stable operation over 168 h, and cascade enrichment to 99.95% of l-phenylalanine optical purity. These results establish interaction–structure coupling as an effective strategy for amplifying subtle molecular recognition into robust transport selectivity in confined membrane systems.

## Abstract 2

Interaction–structure coupling in rGO–ssDNA–SWCNT lamellar membranes amplifies weak chiral recognition into gallery reconfiguration and transport-barrier differences, enabling fast l-Phe permeation with high enantioselectivity.

## Graphical abstract

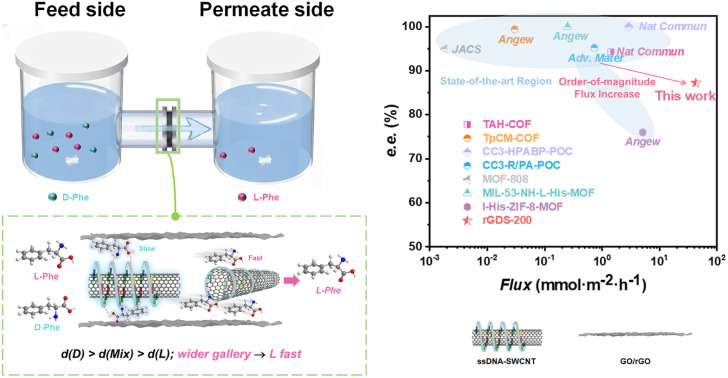

## Introduction

Chirality is a subtle molecular bias with profound practical consequences. In pharmaceuticals, flavors, and agrochemicals, opposite enantiomers can differ sharply in efficacy, odor, metabolism, and toxicity.^1–3^ Yet converting this small stereochemical preference into a continuous, energy-lean separation remains difficult.^4,5^ Membranes are attractive for enantioseparation because they promise low-energy, scalable processing, but most chiral membranes face the same bottleneck: recognition is easier than amplification.^6^ The free-energy difference between enantiomers is typically small and short-ranged. Inside angstrom-confined pathways, diffusion is fast and residence times are short, so weak stereochemical interactions rarely accumulate into a large, persistent difference in transport—especially when the flux is pushed to practical levels.^7,8^

A helpful way to think about this bottleneck is to use a spring-inspired conceptual analogy for structural responsiveness. Here, the spring concept is not intended to describe a quantitatively verified mechanical spring process, nor does it imply directly measured elastic-energy storage or elastic recovery. Instead, it illustrates a more general principle: a weak and local stereospecific interaction may become consequential if it is coupled to a conformationally adaptable interfacial unit that can translate transient molecular recognition into a persistent change in channel geometry and transport resistance. In this sense, angstrom-scale chiral interactions may be amplified into a membrane-level kinetic difference when they trigger reconfiguration of a confined transport pathway.^9^

Turning this idea into a solid-state membrane, however, is not straightforward—especially in lamellar two-dimensional (2D) materials whose transport occurs through sub-nanometre interlayer galleries.^10–14^ The available space is highly confined, making it difficult to introduce a responsive chiral unit without blocking the pathway, generating defects, or disrupting structural continuity across the membrane.^15,16^ This constraint suggests a practical design rule: in 2D nano-galleries, the responsive chiral interfacial unit should be aligned parallel to the sheets. Such an arrangement converts an otherwise difficult 3D anchoring problem into an intercalation-and-alignment strategy that is naturally compatible with lamellar assembly and continuous transport pathways. Recent advances in carbon-based interfacial materials have shown that the physicochemical environment of confined carbon interfaces can be broadly regulated through dimensional integration, surface chemistry, defect engineering, and π-conjugated coupling.^17^ Graphene oxide (GO) and reduced graphene oxide (rGO) lamellar scaffolds provide tunable two-dimensional nano-galleries with adjustable oxygen-containing groups, hydrophilicity, interlayer spacing, and graphitic domains, while one-dimensional single-walled carbon nanotubes (SWCNTs) offer extended π–π-conjugated surfaces that can reinforce interfacial coupling and modulate transport pathways.^18–21^ In particular, regulating oxygen functionalities and defects in GO/rGO can shift the interlayer microenvironment from hydration- or hydrogen-bond-dominated interactions toward stronger π–π interactions and graphitic interlayer coupling.^14^ These advances in functional carbon-based confined interfaces provide the materials basis for our design, in which helically wrapped single-stranded DNA–SWCNT complexes (ssDNA–SWCNT; hereafter referred to as DNA–SWCNT unless otherwise specified) are intercalated into GO/rGO galleries to construct structurally responsive chiral nanochannels.

Building on this carbon-based confined-interface platform, we implement this principle by engineering adaptive chiral nano-galleries in GO/rGO laminates. We intercalate one-dimensional helical DNA–SWCNT complexes into the interlayer space and align them along the galleries, forming a membrane-spanning chiral interface embedded within the confined pathway. Confined between parallel 2D sheets, the helical DNA–SWCNT complexes serve as structurally responsive chiral interfacial modules: enantiomer-specific interactions stereospecifically reconfigure the helix-gallery environment, resetting the interlayer architecture and converting weak chiral recognition into a pathway-scale kinetic barrier split.

In the following, we connect interaction, structural response, and transport kinetics through mutually reinforcing measurements. We show that opposite enantiomers leave distinct post-exposure gallery states, consistent with stereospecific pathway reconfiguration. We quantify kinetic amplification *via* enantiomer-dependent apparent activation barriers, indicating barrier splitting beyond equilibrium adsorption preference. We further provide orthogonal electrochemical signatures after enantiomer soaking that track asymmetric interfacial hindrance. Beyond mechanism, we demonstrate sustained high-flux operation, cascade enrichment, and generality across additional enantiomer pairs, establishing structurally reconfigurable chiral nano-galleries as a transferable design strategy for selective transport.

## Results and discussion

### Design and assembly of GDS membranes

**Scheme 1:**
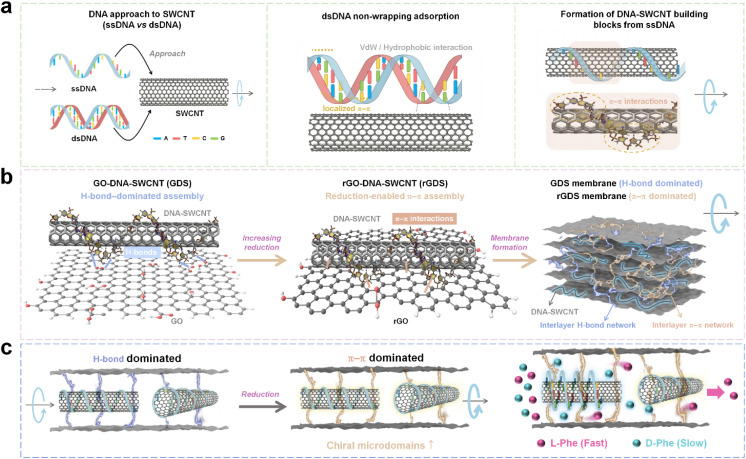
Schematic illustration of the design rationale, assembly route, and enantioselective transport of GDS membranes. (a) Schematic comparison of ssDNA *vs.* dsDNA interactions with SWCNTs: helical wrapping of ssDNA on SWCNT sidewalls *versus* non-wrapping adsorption of dsDNA. (b) Schematic illustration of the fabrication of GDS and rGDS membranes: intercalation of DNA–SWCNT building blocks into GO galleries to form GDS, followed by controlled reduction to obtain rGDS. (c) Schematic illustration of the interlayer coupling modes and enantiomer transport in GDS and rGDS: H-bond dominated and π–π dominated interlayer interaction networks, and the transport disparity between l-phenylalanine (l-Phe) and d-phenylalanine (d-Phe) within the confined interlayer nanochannels.

To implement this concept in a membrane architecture, we used GO laminates as a host because their interlayer galleries form stable, angstrom-scale transport channels.^22–25^ In hydrated GO, however, the channel environment is dominated by fluctuating hydrogen-bond networks,^24,26–28^ which can average out weak stereospecific interactions and limit barrier buildup.^29,30^ Therefore, the key challenge is not confinement itself. Rather, it is the lack of a continuous, membrane-spanning chiral interface within the galleries that can repeatedly engage permeating molecules along the transport pathway and couple chiral asymmetry to the channel–wall interaction field. To address this challenge, we constructed a transferable helical chiral interfacial building block by conformal wrapping of ssDNA on SWCNT sidewalls, enabled by nucleobase π–π interactions^31–33^ (Scheme 1a). Membranes assembled by intercalating DNA–SWCNT building blocks into GO laminates are denoted as GDS. Membranes assembled from pre-thermally reduced GO scaffolds are termed rGDS. Unless otherwise stated, rGDS refers to membranes derived from GO reduced at 50, 150, or 200 °C (Scheme 1b and c).

To prepare the chiral building block, we first denatured double-stranded DNA (dsDNA) to yield a precursor dominated by ssDNA conformations. We selected ssDNA rather than dsDNA for both structural and kinetic reasons.^34,35^ In dsDNA, Watson–Crick base pairing constrains nucleobase accessibility and disfavors continuous π–π contact with SWCNT sidewalls,^34,35^ whereas ssDNA provides conformational freedom for conformal wrapping and sustained interfacial contact.^31^ Denaturation of dsDNA was verified by consistent changes in ultraviolet-visible (UV-vis), Fourier transform infrared (FT-IR), and circular dichroism (CD) spectra (Fig. S1). UV-vis spectroscopy shows a hyperchromic effect around 260 nm, indicating increased base exposure upon strand separation^36^ (Fig. S1a). In the nucleobase-related region (1700–1550 cm^−1^), FT-IR reveals a slight upshift of the band from ∼1692 to ∼1695 cm^−1^ together with an enhanced band at ∼1662 cm^−1^,^37^ indicating altered local nucleobase interaction environments (Fig. S1b). Meanwhile, the characteristic B-form CD couplet is markedly attenuated, reflecting disrupted helical stacking and a more disordered ssDNA-rich conformation^38^ (Fig. S1c). This ssDNA precursor enables stable helical wrapping on SWCNTs.

We then intercalated the DNA–SWCNT helices into GO galleries to assemble the GDS membrane (Scheme 1b). GO sheets are rich in oxygen-containing functionalities.^39^ The interlayer microenvironment is therefore highly polar and capable of extensive hydrogen bonding. Accordingly, the initial interlayer coupling and channel environment in GDS are dominated by hydrated interactions, which provide a permissive yet strongly solvated interface around the embedded helices. To further modulate the interaction type between the channel walls and the embedded building blocks, GDS was converted into rGDS *via* controlled reduction (Scheme 1b). The reduction decreases the density of oxygen sites and restores sp^2^ conjugated domains in rGO.^40^ As a result, π–π interactions and nonpolar interfacial contributions become more prominent, shifting the dominant coupling from hydration-mediated interactions toward stronger graphitic interlayer coupling. This tunable interlayer chemistry allows the same DNA–SWCNT unit to operate under distinct interaction landscapes in GO *versus* rGO laminates, providing a controllable structural platform to probe how the interlayer interaction landscape regulates enantiomer-triggered structural responses and translocation barriers (Scheme 1c).

### Multiscale structural and chemical characterization of GDS membranes

**Fig. 1:**
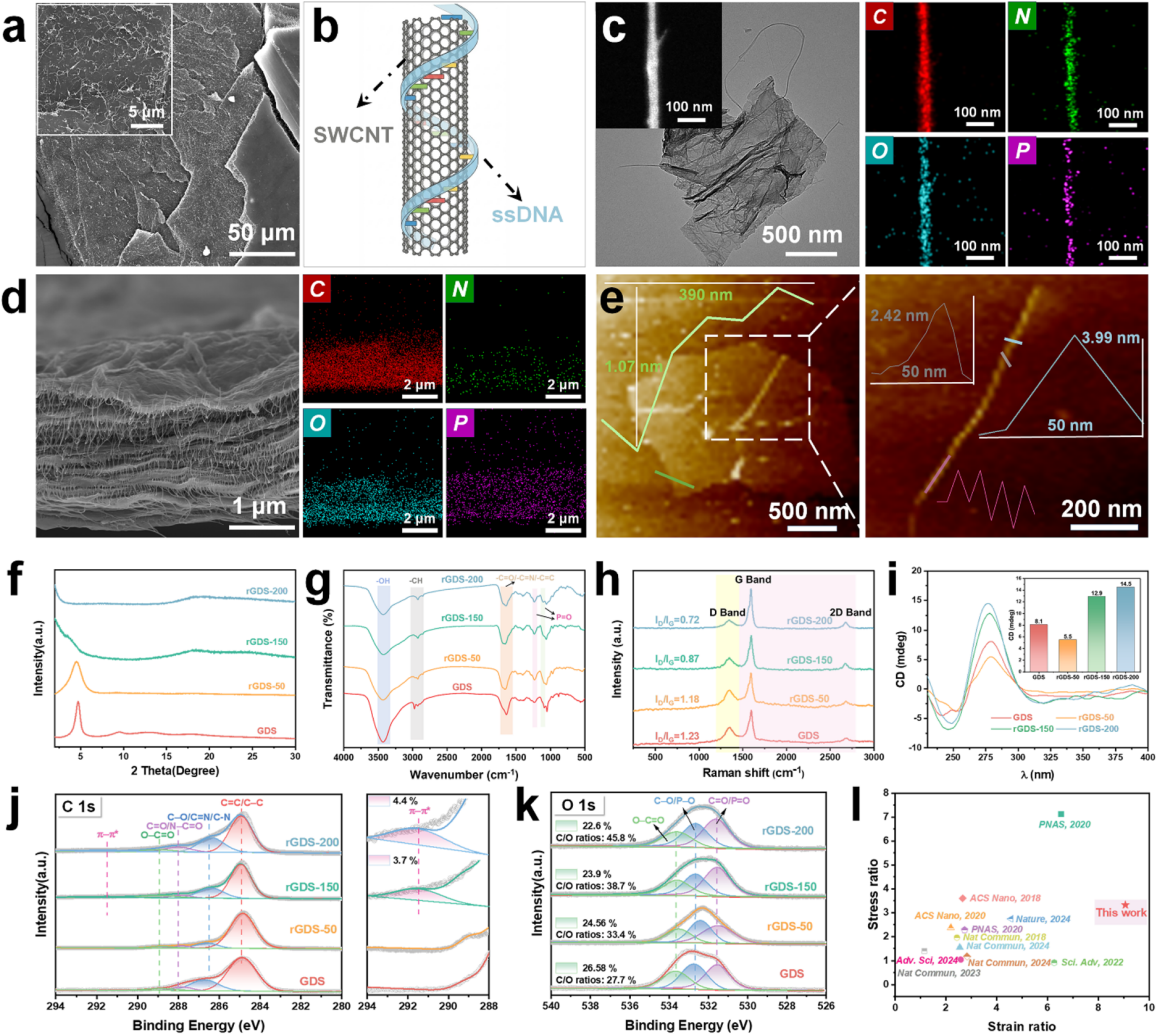
Morphological and structural characterization of GDS/rGDS membranes (GDS: DNA–SWCNT–intercalated GO laminates; rGDS: DNA–SWCNT co-assembled with pre-thermally reduced GO scaffolds; unless otherwise specified, DNA–SWCNT refers to ssDNA–SWCNT). (a) Surface SEM image of a tape-peeled GDS membrane (inset: higher magnification). (b) Schematic illustration of ssDNA helically wrapping the sidewall of a SWCNT. (c) TEM image of the GDS sample and the corresponding EDS elemental maps (C, N, O, and P). (d) Cross-sectional SEM image of the GDS membrane and the corresponding EDS elemental maps (C, N, O, and P). (e) AFM topography and height profiles of the GDS membrane. (f) XRD patterns of GDS and rGDS-50/150/200 membranes, where the number (50/150/200) denotes the GO thermal-reduction temperature (°C) used to prepare the rGO scaffold. (g) FT-IR spectra of GDS and rGDS membranes. (h) Raman spectra of GDS and rGDS membranes. (i) CD spectra of GDS and rGDS membranes (inset: summary of CD amplitude). (j) High-resolution XPS C 1s spectra of GDS and rGDS membranes. (k) High-resolution XPS O 1s spectra of GDS and rGDS membranes. (l) Mechanical-property benchmarking of the rGDS-200 membrane against previously reported membranes.^44–53^

First, rGO dispersions with different reduction degrees were prepared. The dispersions (or pristine GO) were then co-assembled with the DNA–SWCNT building blocks into freestanding membranes using 0.08 MPa negative-pressure filtration, followed by a series of characterization studies to examine their microstructures. Unlike the pristine GO membrane that exhibits a brownish-yellow appearance, the resulting GDS and rGDS membranes display a uniform deep-black color without any visible particle agglomeration on the membrane surface (Fig. S2). Meanwhile, UV-vis spectra of the filtrates exhibit no discernible ssDNA absorption band around ∼260 nm (ref. 36) (Fig. S2), indicating that ssDNA is effectively retained within the interlayer galleries of the membranes with negligible loss during filtration. Scanning electron microscopy (SEM) analyses further confirm the structural integrity of the membranes. Surface SEM images reveal that GD (GO–ssDNA), GS (GO–SWCNT), GDS, and the rGDS series membranes all preserve the typical randomly wrinkled morphology of GO and form continuous, compact surfaces on the micrometer scale, with no observable pinholes or macroscopic cracks (Fig. S3). These results demonstrate good structural reproducibility of the negative-pressure filtration process and confirm that rGDS membranes based on rGO can likewise be fabricated as intact, continuous membranes. To probe the internal stacking features, a tape-peeling method was employed to expose fractured interfaces. Both GDS (Fig. 1a) and rGDS (Fig. S4) exhibit representative step-like interlayer delamination. At a smaller field of view (5 µm scale bar), abundant fibrous networks are observed to bridge across flake boundaries, evidencing that the SWCNT skeleton has been successfully embedded within the matrix and forms an interconnected network. Cross-sectional SEM further supports the presence of a well-defined lamellar architecture (Fig. 1d and S5). Importantly, the cross-sectional distributions of N and P elements in GDS membranes confirm that the DNA component is not merely adsorbed on the surface but is uniformly intercalated throughout the membrane thickness (Fig. 1d).

To verify the ssDNA wrapping configuration on the SWCNT sidewalls (schematically illustrated in Fig. 1b), transmission electron microscopy (TEM) and atomic force microscopy (AFM) characterization experiments were performed. TEM images reveal abundant 1D linear features anchored on the GO nanosheets with morphologies consistent with SWCNTs. Corresponding energy-dispersive X-ray spectroscopy (EDS) elemental mapping shows that the DNA-derived N and P signals are highly co-localized with the linear carbon framework of SWCNTs and distributed continuously or in a bead-like manner along the tube axis, providing direct composition evidence for the formation of DNA–SWCNT building blocks (Fig. 1c). AFM further corroborates these observations: the thickness of the underlying monolayer GO nanosheet is ∼1.07 nm (green trace) (Fig. 1e). Height-profile analysis along an individual SWCNT indicates a thickness of ∼2.42 nm for the exposed segment (gray trace), whereas the wrapped region increases markedly to ∼3.99 nm (blue trace). Moreover, the axial height profile exhibits periodic undulations (pink trace), which is consistent with a helical ssDNA wrapping morphology on the SWCNT surface (Fig. 1e).

Building on the above morphological evidence, we further employed spectroscopic characterization experiments to validate the intercalation of the DNA–SWCNT building blocks from both structural and chemical perspectives, and to compare the oxygen functionalities and carbon-framework signatures between GDS and rGDS. In X-ray diffraction (XRD), the (001) reflection of GO appears at 2*θ* ≈ 10.9° (Fig. S6a). Upon introducing ssDNA or SWCNTs, the (001) peak shifts to ∼6.9° for GD and ∼5.1° for GS, and further to ∼4.7° for GDS, corresponding to an enlarged interlayer spacing and indicating that ssDNA, SWCNTs, and their hybrid building blocks expand the GO interlayer galleries (Fig. S6a). For rGDS membranes, a broad feature emerging in the 2*θ* ≈ 24°–26° range is attributed to the interlayer stacking of reduced rGO domains and the carbonaceous nanostructural framework after reduction (Fig. 1f). Compared with GO, GDS exhibits distinct phosphate-backbone absorptions in the FT-IR spectrum, including P=O (∼1233 cm^−1^) and P–O (∼1060 cm^−1^) bands^23,26^ (Fig. S6b). X-ray photoelectron spectroscopy (XPS) survey spectra and high-resolution analyses further confirm pronounced N 1s (∼399.1 and ∼400.3 eV) and P 2p signals in GD and GDS (Fig. S7a–c). Consistently, the C 1s spectra of GD/GDS resolve contributions associated with N-containing functionalities (*e.g.*, C–N/C=N and N–C=O), while the O 1s spectra can also be deconvoluted into P–O/P=O-related components characteristic of the phosphate backbone (Fig. S7d and e). These evolutions in the carbon/oxygen chemical environments corroborate the N 1s and P 2p signatures, evidencing the effective incorporation of DNA-derived chemical motifs into the composite membranes. Notably, the ssDNA-associated XPS signals (N 1s and P 2p) remain clearly detectable in the rGDS series membranes (Fig. S7f–h), strongly supporting that the DNA–SWCNT units can still be successfully assembled and retained *via* noncovalent interactions even after the GO framework is converted to an rGO scaffold.

By selecting thermally reduced GO scaffolds with different reduction degrees (50–200 °C), we modulated the chemical microenvironment within the interlayer nanochannels. FT-IR spectra show that the –OH stretching band (3200–3600 cm^−1^) progressively weakens with increasing reduction temperature, whereas the phosphate-backbone-related absorptions remain observable (Fig. 1g). XPS quantification corroborates the deoxygenation: the O–C=O component in the O 1s region decreases from 26.58% (GDS) to 22.60% in the deeply reduced rGDS membrane prepared at 200 °C, while the atomic C/O ratio increases from 27.7% to 45.8% (Fig. 1k and S7h). Meanwhile, Raman spectroscopy reveals a two-stage evolution of the carbon framework upon component introduction and subsequent thermal reduction. First, in the GO/GD/GS/GDS comparison, *I*_D_/*I*_G_ changes markedly upon introducing SWCNTs (Fig. S6c and 1h): relative to pristine GO (*I*_D_/*I*_G_ ≈ 1.79), the *I*_D_/*I*_G_ ratios of GS and GDS decrease to ∼1.15 and ∼1.23, respectively, indicating that incorporation of the SWCNT skeleton effectively reduces the defect level of the composite membranes. Subsequent thermal reduction further drives the carbon framework toward a more pronounced sp^2^/π-conjugated character;^41^ for the 200 °C-reduced rGDS membrane, the *I*_D_/*I*_G_ ratio decreases to ∼0.72 (*vs.* ∼1.23 for GDS), accompanied by an intensified 2D band. Consistently, the C 1s spectrum of this deeply reduced rGDS membrane exhibits a distinct π–π* shake-up satellite feature (∼4.4%)^42^ (Fig. 1j). This deoxygenation trend is also reflected in interfacial properties. Zeta-potential measurements show that the precursor dispersion of GDS has a *ζ* potential of ∼−47 mV; with increasing reduction temperature, the absolute *ζ* potential of the rGDS precursor dispersions gradually decreases, reaching ∼−37 mV for the dispersion prepared from GO thermally reduced at 200 °C (Fig. S8a). Meanwhile, the water contact angle of the resulting membranes increases from ∼75° for the mildly reduced rGDS membrane prepared at 50 °C to ∼84° for the deeply reduced membrane prepared at 200 °C (Fig. S8b), indicating reduced wettability and enhanced hydrophobicity upon deeper reduction. Taken together, these results demonstrate that increased reduction leads to fewer oxygenated sites while strengthening sp^2^/π-related spectroscopic signatures, in line with our strategy of tuning the nanochannel microenvironment by controlling the deoxygenation degree of rGO, thereby promoting a shift in the dominant interlayer interactions from a hydrogen-bonding network toward a π–π stacking-dominated mode (Scheme 1b).

Importantly, such structural and chemical modulation is manifested in the CD spectra as distinct chiroptical responses. The CD spectra show a pronounced enhancement in ellipticity after complexing ssDNA with SWCNTs (Fig. S9a), suggesting that the DNA–SWCNT interfacial coupling can amplify the overall chiroptical signal of the system.^43^ This chiral response is retained in the solid-state GDS membrane. Notably, the CD amplitude of rGDS membranes assembled from thermally reduced GO scaffolds (50–200 °C) exhibits a non-monotonic temperature dependence: it decreases from 8.1 mdeg for GDS to 5.5 mdeg after reduction at 50 °C, then increases to 12.9 mdeg after reduction at 150 °C and further to 14.5 mdeg after reduction at 200 °C (Fig. 1i). This trend implies that mild reduction (*e.g.*, GO thermally reduced at 50 °C) may temporarily weaken the CD signal by attenuating the hydrogen-bonding network, whereas deeper reduction with enhanced sp^2^ domains may strengthen interfacial π–π interactions, thereby generating a more confined microenvironment that effectively stabilizes and amplifies the chiral signal.

To evaluate the mechanical impact of introducing DNA and SWCNTs, tensile stress–strain tests were performed on GO, GD, GS, and GDS membranes. Compared with GO, the GS membrane exhibits a markedly elevated stress level, reaching 7.1 MPa, indicating that SWCNT incorporation enhances the load-bearing capability of the membrane. With further introduction of DNA, the GDS membrane delivers an ultimate stress of ∼8.1 MPa with an extended elongation at break of ∼1.7%, suggesting improved ductility upon DNA incorporation (Fig. S9b). Notably, the rGDS membranes assembled from GO thermally reduced at different temperatures exhibit excellent mechanical performance after reduction treatment. The overall stress–strain profiles of these rGDS membranes remain comparable to that of GDS, indicating that the variation in deoxygenation degree does not cause an apparent deterioration in mechanical response. Meanwhile, rGDS assembled from GO thermally reduced at 150 °C and 200 °C shows a slightly higher stress level, with the membrane assembled from GO thermally reduced at 200 °C reaching an ultimate stress of ∼9.8 MPa and an elongation at break of ∼1.8% (Fig. S9c). These results suggest that the reduction-induced enhancement of interlayer π–π stacking strengthens interfacial interactions, thereby further improving the mechanical performance. Benchmarking against representative lamellar composite membranes reported in the literature,^44–53^ the membrane assembled from GO thermally reduced at 200 °C falls into the upper range in terms of stress–strain enhancement relative to GO, supporting its potential for engineering-relevant applications (Fig. 1l and Table S1).

### Chiral separation mechanism of GDS membranes

The chiral separation mechanism of the GDS and rGDS (assembled from thermally reduced GO scaffolds) membranes was elucidated through the investigation of three complementary aspects: the enantiomer-dependent interlayer structural response, apparent activation energy (*E*_a_) disparities quantified *via* temperature-dependent Arrhenius permeation analysis, and interfacial transport kinetics determined by electrochemical measurements.

**Fig. 2:**
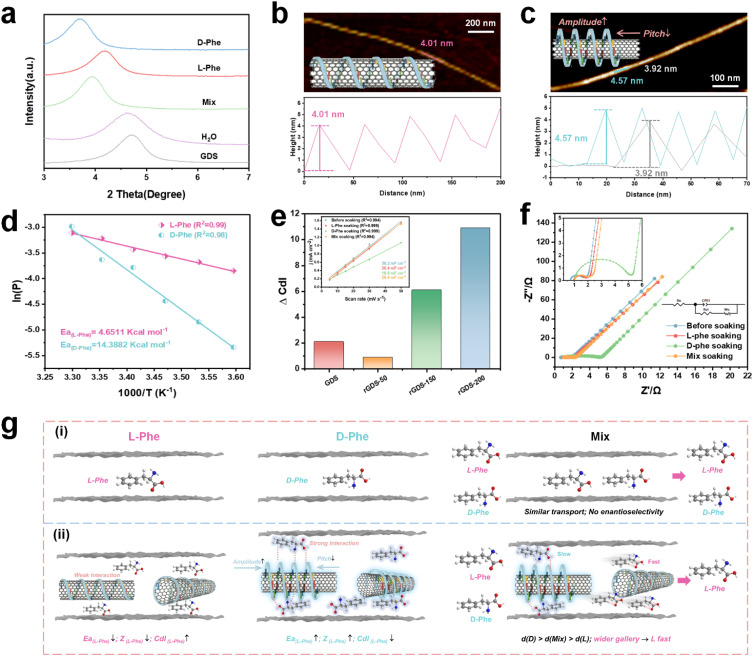
Mechanistic insights into chiral transport in GDS/rGDS membranes. (a) XRD patterns of the GDS membrane after soaking in H_2_O, l-Phe, d-Phe, or racemic (Mix) solutions. (b and c) AFM images and the corresponding height profiles of the DNA–SWCNT chiral building unit after interaction with l-Phe (b) and d-Phe (c). (d) Arrhenius plots for l-Phe and d-Phe permeation across the rGDS-200 membrane (a composite membrane assembled by intercalating ssDNA–SWCNT building blocks into GO scaffolds that have been thermally reduced at 200 °C (rGO)). (e) Enantiomer-induced changes in double-layer capacitance (Δ*C*_dl_) for the GDS and rGDS membranes after pre-soaking in l-Phe or d-Phe, with the inset showing representative *C*_dl_ for rGDS-200 before soaking and after soaking in l-Phe, d-Phe, or Mix. (f) Nyquist plots of rGDS-200 before soaking and after soaking in l-Phe, d-Phe, or Mix, together with the corresponding fitting results. (g) Schematic illustration of the chiral separation mechanism comparing GO (i) and rGDS membranes (ii).

We first examined whether the interlayer nanochannels exhibit an enantiomer-dependent structural response. Upon immersion in water, the GO membrane shows a clear low-angle shift of the (001) reflection, consistent with typical hydration-induced swelling. However, after soaking in l-phenylalanine (l-Phe), d-phenylalanine (d-Phe), or the racemate (Mix), the primary peak shifts slightly further yet remains essentially indistinguishable among the three conditions (Fig. S10). In contrast, the GDS membrane incorporating the chiral DNA–SWCNT building unit displays a pronounced enantiomer-dependent response: the main reflections after exposure to l-Phe, d-Phe, and Mix appear at 2*θ* = 4.20°, 3.70°, and 3.94°, respectively. Based on Bragg's law, the corresponding interlayer spacings are estimated to be *d*(l) ≈ 2.10 nm, *d*(Mix) ≈ 2.24 nm, and *d*(d) ≈ 2.39 nm, respectively, yielding a persistent ordering of *d*(d) > *d*(Mix) > *d*(l) (Fig. 2a).

To elucidate the microscopic origin of the structural disparity, we further characterized the DNA–SWCNT building unit by complementary spectroscopic and morphological analyses. In the FT-IR spectra, compared with l-Phe, d-Phe interacting with DNA–SWCNT gives a more pronounced shoulder variation around ∼1610 cm^−1^ and is accompanied by new absorption features or an intensified band profile near ∼704 and 708 cm^−1^ (Fig. S11a), suggesting that d-Phe engages the chiral DNA–SWCNT unit through an interaction mode distinct from that of l-Phe. AFM provides direct evidence for this chirality-dependent local structural response. After exposure to l-Phe, both the morphology and the height of DNA–SWCNT (∼4.01 nm) remain essentially identical to the pristine state (Fig. 2b and 1e), and no discernible conformational perturbation is observed. By contrast, d-Phe treatment yields a more heterogeneous height profile, where the local thickness increases to ∼4.57 nm and the height trace exhibits larger fluctuations together with a reduced spacing between adjacent characteristic peaks (Fig. 2c). Given the intrinsic rigidity of the SWCNT backbone, these changes are more plausibly attributed to a local conformational rearrangement of the flexible ssDNA wrapping layer induced by d-Phe, which drives the wrapping geometry toward a more compact axial packing. Notably, this microscale interaction disparity is consistent with the macroscopic mechanical response, and membranes soaked in d-Phe display higher tensile stress levels than those treated with l-Phe (Fig. S11b), further supporting a stronger interaction between d-Phe and the chiral building unit.

These structural signatures translate into a large kinetic barrier split that becomes prominent after deep reduction. In GDS, Arrhenius analysis yields only a small difference between the apparent activation energies for l-Phe and d-Phe (Fig. S12). By contrast, in the rGDS membrane assembled from GO thermally reduced at 200 °C, the *E*_a_ of d-Phe increases to ∼14.39 kcal mol^−1^, whereas that of l-Phe remains at ∼4.65 kcal mol^−1^ (Fig. 2d), yielding an energy-barrier difference of ∼9.74 kcal mol^−1^. To probe the origin of this *E*_a_ gap, we pre-soaked the membranes in l-Phe, d-Phe, or racemic solutions and then performed electrochemical characterization on the treated GDS and rGDS membranes. With the reduction-induced growth of sp^2^ domains, the double-layer capacitance (*C*_dl_) increases from 2.9 mF cm^−2^ for GDS to 30.2 mF cm^−2^ after reduction at 200 °C (Fig. S13a), indicating a substantially expanded electrochemically active interface after reduction. More importantly, for the same membrane sample, *C*_dl_ after d-Phe treatment is consistently lower than that after l-Phe treatment, and this difference is most pronounced for the 200 °C-reduced membrane (Fig. 2e and S13b–d). This suggests that d-Phe tends to form a higher-coverage adsorbed layer at the channel interface,^54^ which reduces the effective electroactive area involved in double-layer charging through a shielding effect. Impedance spectroscopy further reveals enantiomer-dependent interfacial resistance. In Nyquist plots, the high-frequency semicircle is commonly associated with the charge-transfer resistance (*R*_ct_) at the membrane–solution interface. Although the overall impedance decreases after reduction (Fig. S14a), enantiomer-dependent differences remain discernible within the same membrane and become clearer as the reduction degree increases (Fig. S14b–d). In the rGDS membrane assembled from GO thermally reduced at 200 °C, d-Phe treatment raises *R*_ct_ to 3.90 Ω, whereas l-Phe and racemic conditions show only minor changes (Fig. 2f and Table S2). Consistently, the cyclic voltammetry (CV) response after reduction at 200 °C is further suppressed under the d-Phe conditions, in line with the electrochemical impedance spectroscopy (EIS) trend (Fig. S15). Collectively, these results support that, within the reduced chiral interface strengthened by π–π interactions, d-Phe more likely forms a denser adsorbed layer that decreases the accessible interface and increases the resistance to interfacial charge transfer and ion migration, which manifests as a higher *E*_a_ and suppressed d-Phe permeation. In comparison, l-Phe perturbs the interfacial electrochemical response to a smaller extent, thereby retaining a lower *E*_a_ and enabling faster permeation.

Taken together, Fig. 2g summarizes the chiral separation mechanism of the rGDS membrane. The pristine GO channels exhibit nonselective diffusion because they lack defined recognition sites (i), whereas within the interlayer galleries of this deeply reduced rGDS membrane (ii), d-Phe interacts more strongly with the DNA–SWCNT chiral unit and induces local conformational adjustment of the flexible ssDNA wrapping layer. This interaction-driven adjustment further leads to an enantiomer-dependent gallery response. On the one hand, at the deeply reduced π–π-dominated interface, the stronger d-Phe interaction, evidenced by a decreased *C*_dl_ and an increased *R*_ct_, is associated with a substantially elevated activation energy for transmembrane permeation, thereby suppressing d-Phe transport through the galleries. On the other hand, under racemic conditions, *d*(Mix) > *d*(l) indicates that d-Phe shifts the interlayer configuration toward a more expanded state. Importantly, this d-Phe-induced gallery expansion should not be interpreted as a simple enlargement of a free diffusion channel for d-Phe itself. Rather, the expanded gallery state originates from the stronger d-Phe–interface interaction and the resulting rearrangement of the flexible wrapping layer. Therefore, geometric channel opening and interfacial hindrance contribute differently to transport: the former provides a more open lamellar environment, whereas the latter selectively raises the effective migration barrier of d-Phe. In this coupled state, d-Phe remains strongly hindered at the chiral interface, whereas l-Phe can permeate more rapidly through the more open galleries with a lower activation energy, ultimately enabling chiral sieving. These two effects are synergistic rather than contradictory: the stronger d-Phe–interface interaction increases the migration resistance of d-Phe itself, while the same interaction triggers gallery reconfiguration that provides a more open transport environment for faster l-Phe permeation under racemic conditions. It should be noted that this interaction–structure coupling mechanism is distinct from a conventional chiral site–enantiomer adsorption-recognition mechanism. In conventional affinity-based chiral membranes, selectivity is typically attributed to differences in static binding or adsorption affinity between fixed chiral sites and two enantiomers. In the present rGDS system, enantiomer-specific recognition is the initial event rather than the sole origin of selectivity. The weak stereospecific interaction at the flexible DNA–SWCNT interface is further translated into local wrapping-layer perturbation, lamellar gallery reconfiguration, and interfacial transport resistance. Thus, the membrane-level selectivity arises from a coupled sequence of molecular interaction, structural response, and transport-barrier differentiation, rather than from simple retention of one enantiomer at isolated adsorption sites. This distinction explains how subtle l-/d-Phe recognition can be amplified into a sustained kinetic barrier difference while maintaining high-flux transport through the lamellar galleries. In this way, the continuous rGO lamellar galleries support fast molecular transport, while the responsive DNA–SWCNT chiral interface provides enantiomer-dependent interfacial resistance rather than global pore narrowing or nonspecific strong adsorption. This separation of high-flux transport pathways and selective barrier differentiation helps alleviate the conventional flux–selectivity trade-off in chiral membrane separation.

### Chiral separation performance of GDS membranes

**Fig. 3:**
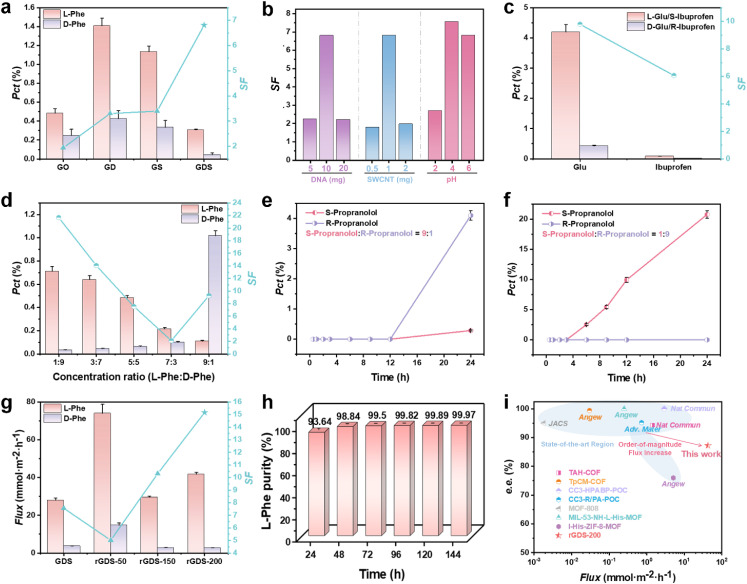
Chiral separation performance of GDS/rGDS membranes. (a) Pct of l-Phe/d-Phe and the maximum SF for GO, GD, GS, and GDS membranes, where Pct is taken at the time point when SF reaches its maximum for each membrane (l-Phe and d-Phe were tested at the same initial concentration of 0.018 M). (b) Effects of ssDNA loading, SWCNT loading, and driving-solution pH on the separation of l-Phe/d-Phe by the GDS membrane (l-Phe and d-Phe were tested at the same initial concentration of 0.018 M). (c) Permeation behavior of the GDS membrane under single-enantiomer feeds of l-Glu and d-Glu, and *S*-ibuprofen and *R*-ibuprofen (*C* = 0.018 M). (d) Phe permeation under non-equimolar mixed feeds with varied l : d ratios (from 1 : 9 to 9 : 1) at a constant total concentration (*C*_tot_ = 0.018 M). (e and f) Permeation kinetics of propranolol under non-equimolar mixed feeds with *S* : *R* = 9 : 1 (e) and *S* : *R* = 1 : 9 (f) at *C*_tot_ = 0.018 M. (g) Flux and the maximum SF of GDS and rGDS-*T* (*T* = 50, 150, or 200 °C) membranes under equimolar racemic Phe (l-Phe : d-Phe = 1 : 1 and *C*_tot_ = 0.018 M), where the flux is taken at the time point when SF reaches its maximum for each membrane. (h) Purification of l-Phe/d-Phe using the rGDS-200 membrane. (i) Comparison of e.e.–flux performance between the rGDS-200 membrane and representative chiral separation membranes.^40,58–65^

To systematically evaluate the chiral separation performance of the GDS and rGDS membranes, the as-prepared membranes were mounted in a custom-built permeation device and examined by permeation experiments (Fig. S16). For single-enantiomer feeds, solute accumulation in the right-side driving solution was quantified by UV-vis spectroscopy, whereas for racemic or non-equimolar feeds, enantiomeric composition and enantiomeric excess (e.e.) were determined by chiral high-performance liquid chromatography (HPLC) (SI Methods). To establish the pivotal role of the chiral building units in the separation process, we compared the enantioselective transport of l-Phe and d-Phe across the GDS membrane with that across a series of control membranes including GO, GD, and GS. The GDS membrane exhibited a separation factor (SF) that was substantially higher than those of all control groups (Fig. 3a and S17). Specifically, pristine GO showed nonselective and nonspecific permeation, whereas the GS membrane (lacking ssDNA) or the GD membrane (lacking SWCNTs) displayed only marginal enantiomer discrimination. This performance contrast indicates that the cooperative assembly of ssDNA and SWCNTs is required to achieve pronounced enantiomeric differentiation. Additional GO–dsDNA–SWCNT control experiments further confirmed the importance of flexible ssDNA wrapping: replacing ssDNA with un-denatured dsDNA under otherwise identical assembly and testing conditions led to only weak l-/d-Phe discrimination, with SF values of 1.02–1.60, much lower than that of the GDS membrane (Fig. S17e). This result indicates that the high enantioselective transport does not simply arise from the co-assembly of DNA, SWCNTs, and GO, but is closely associated with the flexible and continuous chiral interface formed by ssDNA wrapping on SWCNTs. SWCNTs likely contribute to establishing a continuous, through-thickness scaffold that preserves channel connectivity,^55^ while ssDNA encodes chiral information at the interface and provides differentiated interaction sites for the two enantiomers.^56^ The absence of either component compromises the continuity and effectiveness of the chiral interfacial landscape, thereby leading to a pronounced loss of selectivity.

Building on these results, we further examined how the component loadings govern separation performance. We found that, throughout the permeation period, the permeation percentages (Pct) of l-Phe were consistently higher than those of d-Phe, and the GDS membrane delivered the best separation efficiency when the SWCNT loading was 1 mg and the ssDNA loading was 10 mg (Fig. 3b, S18 and S19). Kinetic analysis indicates opposite regulation trends of ssDNA and SWCNTs on transport, suggesting that these two components play distinct structural roles in the interlayer microenvironment (Fig. S18 and S19). Specifically, increasing the ssDNA loading from 5 mg to 20 mg continuously reduced the overall Pct values of l-Phe and d-Phe (Fig. S18), consistent with a crowding-induced barrier in confined galleries, where dense nucleic-acid chains impose steric and volume exclusion and thereby hinder diffusion.^53^ Meanwhile, the separation factor (SF) reached a maximum at 10 mg ssDNA, and further loading did not improve selectivity but instead likely suppressed separation due to site crowding or reduced accessibility of effective recognition interfaces (Fig. S18). In contrast, at a fixed ssDNA loading of 10 mg, increasing the SWCNT content enhanced the overall Pct, indicating that the 1D rigid scaffold helps maintain through-thickness channel continuity and effective transport interfaces (Fig. S19). However, when the SWCNT loading increased to 2 mg, SF did not increase accordingly and even declined despite the higher flux, implying that excessively rapid transport can shorten the effective contact time between permeating molecules and the chiral interface, which is unfavorable for fine enantiomer discrimination (Fig. S19). Therefore, the combination of 1 mg SWCNTs and 10 mg ssDNA provides an optimal balance between Pct and selectivity. The pH of the driving solution was also identified as a key variable for tuning separation (Fig. 3b and S20). As the pH of the right-side driving solution decreased from 6 to 2, the larger transmembrane proton gradient increased the driving force and raised Pct. However, the pH effect is not limited to proton-gradient-driven transport. Proton back-diffusion from the driving side may partially acidify the interfacial region near the membrane and alter the local charge state of Phe, especially by changing the carboxylate/carboxylic-acid balance under strongly acidic conditions. Such changes may modify the hydrogen-bonding and electrostatic interaction patterns between Phe and the ssDNA–SWCNT chiral interface. In addition, the increased H^+^ concentration may screen the local charges around oxygen-containing groups and the ssDNA phosphate backbone, thereby perturbing the interfacial interaction field required for stereospecific recognition. Under strongly acidic conditions at pH 2, possible protonation or conformational perturbation of the ssDNA wrapping layer may further weaken the translation of enantiomer-specific interactions into gallery reconfiguration and transport-barrier splitting. Therefore, decreasing pH can enhance flux by increasing the driving force, but excessive acidity may reduce selectivity by disturbing the molecular charge state and the chiral interfacial environment. Considering these factors together, we selected pH 4 as the optimal condition, under which the GDS membrane achieved an SF of 7.6 for l-Phe and d-Phe. To evaluate the generality of this strategy, we extended the test set to chiral molecules with different physicochemical properties, including an amino acid, glutamic acid, and drug molecules, ibuprofen, propranolol, and 2-methylpiperazine (Fig. 3c and S21). The GDS membrane exhibited strong selectivity for glutamic acid (SF ≈ 9.8) and ibuprofen (SF ≈ 6.1), whereas the separation of propranolol and 2-methylpiperazine was comparatively weaker. These differences suggest that substrate-dependent molecular features influence the separation outcome. Molecules capable of forming moderate and distinguishable interfacial interactions with the rGDS galleries appear to be more favorable for this membrane system. Aromaticity and moderate hydrophobicity may enhance π–π and hydrophobic interactions with the π-conjugated interface, while charge state and hydrogen-bonding ability may further affect the interaction with the ssDNA wrapping layer. In contrast, excessively weak interactions may lead to nearly nonselective diffusion, whereas overly strong retention or nonspecific electrostatic adsorption may mask subtle stereospecific differences and reduce flux. A plausible explanation is that strongly cationic substrates (propranolol and 2-methylpiperazine) can undergo pronounced electrostatic adsorption onto the negatively charged galleries, which promotes interfacial accumulation and site saturation and shifts transport toward an adsorption-controlled regime.^57^ Such nonspecific trapping can obscure subtle stereochemical differences and hinder enantiomer migration. In contrast, glutamic acid and ibuprofen are less prone to strong cationic trapping under these conditions, which reduces nonspecific adhesion and allows chiral-channel interactions to play a larger role in recognition and sieving.

Considering that enantiomer concentrations in practical industrial separations are often non-equimolar and far from equilibrium, we further investigated how feed composition influences separation behavior. Using Phe as a model, we kept the total concentration constant and varied only the l : d ratio. When l : d was adjusted from 1 : 9 to 9 : 1, the Pct of l-Phe decreased as its fraction increased, which is consistent with preferential occupation of a finite number of interaction sites within the channels by the more concentrated enantiomer and the resulting self-blocking that raises its effective migration resistance (Fig. 3d). Notably, in non-equimolar mixtures, competitive transport can markedly amplify membrane selectivity. For propranolol, when the feed *S* : *R* ratio was 9 : 1, the Pct of the *R* enantiomer gradually increased with time and the separation factor of the GDS membrane rose to 14.4 (Fig. 3e). When the composition was reversed to *S* : *R* = 1 : 9, the membrane exhibited near-quantitative retention of the *R* isomer, approaching 100% (Fig. 3f). The same trend was observed for 2-methylpiperazine, Glu, and ibuprofen (Fig. S22), indicating that this membrane is effective not only for resolving equimolar enantiomer pairs but also for upgrading optical purity from composition-biased crude mixtures.

Building on these results, we benchmarked GDS against rGDS membranes derived from GO thermally reduced at 50–200 °C under equimolar racemic Phe feeds, aiming to elucidate how reduction jointly regulates transport kinetics and selectivity. As the reduction became more pronounced, the Pct of l-Phe increased progressively (Fig. S23), suggesting that deoxygenation and restoration of sp^2^-conjugated domains can lower the average mass-transfer resistance within the nanochannels and improve channel connectivity. In contrast to the monotonic increase in l-Phe Pct, the membrane separation factor exhibited a decrease followed by a rise as the reduction depth increased. Specifically, the SF of the GDS membrane was 7.39, which dropped to 3.00 after 50 °C reduction, and then increased to 11.0 and 12.59 after 150 °C and 200 °C reduction, respectively (Fig. 3g and S23). This trend suggests that mild reduction may perturb the original hydrophilic interaction field and interfacial coupling without yet establishing a stable and percolated chiral interaction environment, thereby diminishing enantioselectivity. By contrast, deep reduction in rGDS assembled from GO thermally reduced at 150 °C and 200 °C is more likely to enhance interlayer coupling and reshape the channel microenvironment, amplifying the transmembrane barrier disparity between enantiomers and thus substantially improving selectivity (Fig. 3g and S23). Notably, GDS showed a higher SF at the early stage of permeation around 2 h, followed by a gradual decline, whereas the SF of rGDS assembled from GO thermally reduced at 150 °C and 200 °C membranes increased overall with time and reached higher values at 24 h (Fig. S23). This difference indicates that the interlayer channels after deep reduction better preserve the structural and chiral interfacial microenvironment in aqueous media over extended operation, enabling continuous accumulation and time-scale amplification of enantiomeric disparities (Fig. S23). This reduction-dependent behavior further indicates that the role of thermal reduction is not merely to increase channel hydrophobicity or decrease the average mass-transfer resistance. Instead, reduction reshapes the interlayer interaction field of the lamellar membrane. In less reduced GO/rGDS membranes, residual oxygen-containing groups and interlayer water create a more hydration-dominated interface, where swelling, hydration interactions, and weak graphitic coupling may obscure the subtle stereospecific interaction between Phe and the ssDNA–SWCNT chiral unit. Under such conditions, local chiral recognition is less efficiently translated into a persistent gallery response and transport-barrier splitting. In contrast, the rGDS membrane assembled from GO thermally reduced at 200 °C contains more graphitic domains and provides a more π–π-dominated interfacial environment with stronger graphitic interlayer coupling. This interfacial field strengthens the coupling among the ssDNA–SWCNT unit, aromatic Phe molecules, and the rGO galleries, allowing the stronger d-Phe–interface interaction to more effectively induce local wrapping-layer rearrangement and lamellar gallery reconfiguration. Therefore, the superior selectivity of the rGDS membrane assembled from GO thermally reduced at 200 °C arises not simply from faster diffusion through a more hydrophobic channel, but from the amplified conversion of stereospecific interfacial interactions into a larger apparent transport-barrier difference.

Toward practical deployment, we further evaluated the reusability and enrichment capability of the membrane. After seven cycles of continuous operation over 168 h, the rGDS membrane assembled from GO thermally reduced at 200 °C exhibited a stable enantiomeric excess of 84.6–87.3%, while the l-Phe flux remained at 41.1–43.8 mmol m^−2^ h^−1^ and the d-Phe flux remained at 2.9–3.5 mmol m^−2^ h^−1^, demonstrating robust operational stability and good regenerability (Fig. S24). SEM characterization after 168 h operation further showed that the membrane retained a continuous surface morphology and a discernible lamellar cross-sectional structure, without obvious large cracks, pinholes, or severe delamination (Fig. S25). A six-stage cascade using the rGDS membrane assembled from GO thermally reduced at 200 °C further increased the e.e. of l-Phe to 99.95% (Fig. 3h). Notably, compared with some membrane materials that exhibit peak separation only within short test windows,^36,55–62^ the rGDS membrane assembled from GO thermally reduced at 200 °C reaches its optimal performance at 24 h and sustains high e.e. during continuous seven-day operation while delivering an order-of-magnitude higher flux under the present testing conditions, highlighting its potential for continuous chiral purification (Fig. 3i).

## Conclusions

We show that high-flux enantioselective transport can be achieved by coupling weak stereospecific recognition to structural reconfiguration within lamellar nano-galleries. By intercalating helical ssDNA–SWCNT complexes into GO/rGO laminates, we constructed structurally responsive chiral interfaces that translate enantiomer-specific interactions into persistent gallery responses and differentiated transport barriers. The convergence of enantiomer-dependent interlayer spacing, local wrapping-layer perturbation, Arrhenius-type activation-barrier splitting, and electrochemical interfacial hindrance supports an interaction–structure coupling mechanism rather than a simple static adsorption-recognition process. In this mechanism, d-Phe induces stronger interfacial hindrance, while the resulting gallery reconfiguration helps sustain a kinetic barrier difference that enables faster l-Phe transport. The rGDS membrane therefore combines high flux, stable enantioselectivity, and cascade enrichment capability. Beyond this specific Phe separation system, this work suggests a general design concept for constructing reconfigurable chiral nanochannels by integrating a conformationally responsive 1D chiral unit into a stable 2D lamellar scaffold. An effective 1D chiral-unit-in-2D gallery strategy should balance flexible chiral-interface responsiveness, continuous confined transport pathways, and moderate yet distinguishable substrate–interface interactions, so that subtle molecular recognition can be amplified into membrane-scale selective transport.

## Author contributions

S. H., F. Y., W. H., and Z. L. conceived the project. H. C. and Z. L. supervised the project. S. H., F. Y., W. H., and X. C. performed the experiments and characterization studies. S. H., F. Y., W. H., H. C., H. W., X. C., and Z. L. co-wrote the manuscript. All authors discussed the results and commented on the manuscript.

## Conflicts of interest

The authors declare no conflict of interest.

## Supplementary Material

SC-OLF-D6SC03058K-s001

## Data Availability

All data generated or analysed during this study are included in this article and its supplementary information (SI). Supplementary information: experimental details, material and membrane preparation procedures, characterization methods, permeation and chiral HPLC analysis protocols, transport-parameter calculations, Arrhenius and electrochemical measurement methods, supplementary figures, and supplementary tables. See DOI: https://doi.org/10.1039/d6sc03058k.
