# Supplementary material for: Interaction–structure coupling enables high-flux enantioselective transport in lamellar membranes
Source: Chem Sci. 2026 Jun 19;17(28):13804–15. doi: 10.1039/d6sc03058k (PMC13281870; doi:10.1039/d6sc03058k)
Supplement: SC-017-D6SC03058K-s001 [file SC-017-D6SC03058K-s001.pdf]

Supplementary Information (SI) for Chemical Science.
This journal is © The Royal Society of Chemistry 2026

Supplementary Information

Interaction-Structure Coupling Enables High-Flux Enantioselective Transport in Lamellar Membranes

Siqi Han[1,3], Furong Yuan[1,3], Honghong Cao[2], Wentao Han[1,3], Hongyan Wan[1,3], Ximeng Chen[1,3], Zhan Li[1,3,4,*]

[1]MOE Frontiers Science Center for Rare Isotopes, Lanzhou University, Lanzhou 730000, China.

[2]School of Accounting, Lanzhou University of Finance and Economics, Lanzhou 730020

[3]School of Nuclear Science and Technology, Lanzhou University, Lanzhou 730000, China.

[4]School of Chemistry and Chemical Engineering, Qinghai Nationalities University, Xining 810007, China

*Correspondence: liz@lzu.edu.cn (Z. L.)

Supplementary Text

Materials. Sulfuric acid (H_2SO_4) (96-98 wt%) was purchased from Sichuan Longxi Science and Technology Company. Hydrochloric acid (HCl) (36-38 wt%) and potassium permanganate ($KMnO_4$) were sourced from Chengdu Colony Chemicals Co. Ltd. Graphite powder was obtained from Aladdin Chemical Reagent Company; sodium nitrate ($NaNO_3$) and Hydrogen peroxide (H_2O_2) (30 wt%) were procured from Tianjin Damao Chemical Reagent Factory; Polyvinylpyrrolidone was purchased from Shanghai McLean Biochemical Science and Technology Co. Ltd., PBS Buffer Solution (pH=7.4, containing 137 mM NaCl) was purchased from Wuhan Servicebio Technology Co. Ltd., deoxyribonucleic acid (from salmon sperm), Urea (99.5%), single-walled carbon nanotubes (96%), L-phenylalanine (L-Phe, 99%), D-phenylalanine (D-Phe, 98%), L-glutamic acid (L-Glu, 99%), D-glutamic acid (D-Glu, 98%), (R)-(-)-Ibuprofen (98%) and (S)-(+)-Ibuprofen (98%), (R)-(+)-Propranolol Hydrochloride (R-PPL, 98%), and (S)-(-)-Propranolol Hydrochloride (S-PPL, 98%) were purchased from Shanghai Aladdin Biochemical Technology Co. Ltd. (R)-(-)-2-Methylpiperazine (98%), and (S)-(+)-2-Methylpiperazine (98%) were purchased from Shandong Keyuan Biochemical Co. Ltd. All reagents used in the experiments were analytically pure reagents and were not purified before use. The experimental water used was ultrapure water with a resistance of 18.25 MΩ/cm.

Material characterization. The surface morphology of the synthesized materials was analyzed using a Thermo Scientific Apreo S scanning electron microscope (SEM), which included detailed elemental mapping analysis. Microstructural characterization and determination of chemical compositions were conducted using a transmission electron microscope (TEM, FEI Tecnai G2 F30, USA) operating at an accelerating voltage of 200 kV. Atomic force microscopy (AFM) images were obtained on the Bruker Dimension Icon (Germany) to study the surface roughness of the prepared membrane materials. Chemical structure and coordination environments were elucidated using Kratos AXIS Ultra DLD X-ray photoelectron spectroscopy (XPS) calibrated with a 284.8 eV C1s peak. The crystalline structure and phases of the materials were analyzed using a Rigaku Ultima IV diffractometer, irradiating samples

with Cu Kα radiation at 1.541871Å. For post-soaking XRD measurements, the GDS membranes were first immersed in H2O, L-Phe, D-Phe, or racemic solutions. After soaking, the membranes were removed from the solution and air-dried under ambient conditions before XRD measurement. Therefore, the XRD patterns of the soaked membranes correspond to a post-soaking air-dried state, rather than an in situ wet transport state or a real-time operando hydrated gallery state during permeation. The same post-soaking air-drying procedure was also used for the GO control membranes shown in Fig. S10. Raman spectroscopic analysis was performed with a Rigaku instrument. The NEXUS 670 Fourier Transform Infrared Spectrometer (FT-IR) was utilized to analyze the synthesized materials across wavelengths ranging from 400-4000 cm^{-1} for the various functional groups in their chemical composition. Mechanical properties were characterized by an electrical universal material testing machine (HFT-100, Jinan Hengfa Instrument Co., Ltd.) with a loading rate of 5 mm min^{-1}. The concentration of amino acids in the driving solution was determined using a UV-Vis spectrophotometer (Shanghai Pris Technology Co., Ltd., Model 1901PC) and high-performance liquid chromatography (HPLC, Agilent 1260). Circular dichroism (CD) measurements were conducted using a spectrophotometer (J1500, JASCO, Japan).

Preparation of Graphene Oxide (GO). GO was prepared using the modified Hummers method: Initially, 5 g of graphite powder was weighed and added to a 500 mL three-necked flask along with 5 g of $NaNO_3$. Then, 200 mL of concentrated H_2SO_4 was slowly added in an ice bath over 1h. Subsequently, 20 g of $KMnO_4$ was added slowly at 200 rpm, and the reaction proceeded in a water bath at 38°C for 26 h. Afterward, 250 mL of deionized water was added dropwise at 300 rpm, followed by another 250 mL under the same conditions. The mixture was then transferred to a water bath at 84°C for 15 min. Once the reaction was complete, the solution was poured into a 1000 mL beaker containing 500 mL of deionized water. To complete the process, 30% H_2O_2 was added dropwise until no bubbles were visible in the solution, indicating the reaction's end. The resulting solution was centrifuged to obtain a solid product, which was subsequently washed with 10% HCl solution to remove unreacted ions. After washing with deionized water to neutral pH, the GO was stored at room temperature

for future use. The concentration of GO aqueous dispersion was 6.67 g/L.

Preparation of reduced graphene oxide (rGO). Reduced graphene oxide (rGO) was prepared by a thermal reduction of an aqueous GO dispersion. A predetermined volume of the as-prepared GO dispersion was transferred into a Teflon-lined stainless-steel autoclave, sealed, and heated in an oven at 50, 150, or 200 °C for 3 h. After the reaction, the autoclave was allowed to cool naturally to room temperature. The resulting dispersion was then freeze-dried to afford rGO powders, denoted as rGO-50, rGO-150, and rGO-200 according to the reduction temperature.

Preparation of ssDNA-SWCNT (DNA-SWCNT) complexes. Salmon sperm DNA was dissolved in PBS buffer (pH 7.4, containing 137 mM NaCl) supplemented with 8 M urea to denature double-stranded DNA. The solution was heated to 90 °C under stirring for 1 h. The DNA concentration was adjusted to 5, 10, or 20 mg/mL. After denaturation, the DNA solution was transferred into a dialysis bag and dialyzed against deionized water at room temperature to remove urea and salts, affording an aqueous ssDNA solution. SWCNT were dispersed in 10 mL of an aqueous polyvinyl pyrrolidone (PVP) solution (0.5 wt%) and sonicated to obtain a stable SWCNT dispersion. The ssDNA solution was then added dropwise into the SWCNT dispersion under stirring and the mixture was stirred for 2 h to form the ssDNA-SWCNT (DNA-SWCNT) complex dispersion.

Membrane fabrication

GO membrane: A GO membrane was prepared by vacuum filtration. Specifically, 0.75 mL of GO dispersion containing 5 mg GO was diluted to 10 mL with deionized water and sonicated to ensure homogeneous dispersion. The suspension was vacuum-filtered at 0.08 MPa onto a polyethersulfone (PES) microfiltration substrate (diameter 50 mm, pore size 0.22 μm). The obtained GO membrane is denoted as GO.

GD membrane: For the GD membrane, an ssDNA aqueous solution containing 10 mg ssDNA was diluted to 10 mL. A GO dispersion containing 5 mg GO was then added into the ssDNA solution and the mixture was stirred for 2 h. The resulting suspension was vacuum-filtered under the same conditions as above to yield the GD membrane, denoted as GD.

GS membrane: For the GS membrane, a GO dispersion containing 5 mg GO was added into the SWCNT dispersion containing 1 mg SWCNT and stirred for 2 h. The mixture was subsequently vacuum-filtered under the same conditions to yield the GS membrane, denoted as GS.

GDS membrane: For the GDS membrane, a GO dispersion containing 5 mg GO was added into the DNA-SWCNT complex dispersion and stirred for 2 h. The mixture was vacuum-filtered under the same conditions to obtain the GDS membrane, denoted as GDS. To optimize the separation performance, a series of GDS membranes were prepared by varying the mass loadings of ssDNA and SWCNT while keeping the other fabrication parameters unchanged.

GO-dsDNA-SWCNT control membrane: To further examine the role of ssDNA wrapping, a GO-dsDNA-SWCNT control membrane was prepared under the same conditions as the GDS membrane, except that un-denatured dsDNA was used instead of ssDNA. Specifically, the dsDNA solution was directly mixed with the SWCNT dispersion without urea-assisted denaturation, followed by addition of the GO dispersion and vacuum-assisted filtration under the same conditions used for GDS membrane fabrication.

rGDS-T membrane: Reduced GDS membranes were fabricated by replacing GO with thermally reduced graphene oxide. Specifically, rGO dispersions (rGO-50, rGO-150, and rGO-200) were separately added into the DNA-SWCNT complex dispersion containing 10 mg ssDNA and 1 mg SWCNT, followed by stirring for 2 h. The resulting mixtures were vacuum-filtered under the same conditions to yield rGDS-50, rGDS-150, and rGDS-200 membranes, respectively.

Separation experiments and calculation of transport parameters

Membranes prepared under different conditions were mounted in a custom-built permeation cell (Fig. S16). Unless otherwise specified, permeation tests for performance screening and condition optimization were carried out using single-enantiomer feeds. Specifically, 50 mL of enantiomeric solution was added to the left side, and an equal volume of deionized water was added to the right side as the driving fluid to perform osmotic kinetic experiments at room temperature and rotor stirring was

added to both sides of the separation device. Samples (1 mL each) were taken from both sides at specific time intervals, and the absorbance of enantiomers in the samples was measured by UV spectrophotometer to obtain the concentration from the standard curve. The effective membrane area exposed to permeation was 7.85 × 10^{-5} m^2 (0.785 cm^2), and this value was used as A in the flux calculation. For time-dependent permeation experiments, samples were collected at 0.5, 1, 2, 3, 6, 9, 12, and 24 h. For the cycling stability test, each cycle lasted 24 h, and the permeation performance was evaluated once per cycle.

The permeation percentage was calculated as[1]

$$Pct = \frac{C_t}{C_0} \times 100\%\#() \quad 1.$$

Where Pct represents the permeation percentages, C_t is the solute concentration in the permeate chamber at time t, and C_0indicates the original concentration (in g/L).

For a pair of opposite enantiomers tested under identical operating conditions, the separation factor was calculated as[2]

$$SF = \frac{Pct_1}{Pct_2}\#(2)$$

where $Pct_1$and Pct_2 are the permeation percentages of the faster- and slower-permeating enantiomers, respectively.

The Flux ($mmol \cdot m^{-2} \cdot h^{-1}$) was calculated by[3]

$$Flux = \frac{n}{A \times T}\#(3)$$

In this equation, *n* represents the mass of material passing through the membrane (mmol), *A* represents the effective area of the membrane during separation (m^2), and *T* represents the sampling time (h).

For experiments where both enantiomers coexist in the same sample, including racemic feeds, long-term cycling, and cascade purification, the permeate was analyzed by chiral HPLC equipped with a CROWNPAK CR(+) column. The mobile phase was aqueous $HClO_4$ adjusted to pH 1.5 mixed with methanol at 85/15 (v/v), and the column temperature was maintained at 35 °C. Enantiomeric excess was calculated from the chromatographic peak areas as

$$e.e.\% = \frac{A_L - A_D}{A_L + A_D} \times 100\%\#\#\#(4)$$

where $A_L$and $A_D$are the peak areas of the two enantiomers.

Transport energy barriers measurement

Measurements were carried out in a thermostatic water bath with magnetic stirring set at 5, 10, 15, 20, 25, and 30°C. The energy barriers (Ea) for molecules to cross the membrane can be calculated using an Arrhenius-type Equation

$$ln(P) = ln(\alpha) - \left(\frac{E_a}{R} \cdot \frac{1}{T}\right) \quad (5)$$

where α is the exponential prefactor, R (1.985×10^{-3} kcal mol^{-1} K^{-1}) is the gas constant, T (K) is the temperature, and E_a (kcal mol^{-1}) is the apparent activation energy. The molecular flux P (mol m^{-2} h^{-1}) at each temperature was used to construct Arrhenius plots of $ln(P)$versus $1/T$. Linear fitting of the Arrhenius plots yielded a slope of $-E_a/R$, from which $E_a$was calculated.

Electrochemical measurements

Electrochemical measurements. Electrochemical measurements were performed on a CHI760E electrochemical workstation using a three-electrode configuration. The membrane was mechanically pressed onto nickel foam, and the membrane/nickel foam assembly was used as the working electrode. An Ag/AgCl electrode and a platinum plate were used as the reference electrode and counter electrode, respectively. The electrolyte was 0.1 M KCl. Before electrochemical measurements, the membranes were pre-soaked in L-Phe, D-Phe, or racemic solution for 12 h, with the total solute concentration of each pretreatment solution fixed at 3 g L^{-1}. The double-layer capacitance (Cdl) was determined from cyclic voltammetry (CV) curves recorded at scan rates of 5, 10, 15, 20, 30, and 50 mV s−1. The capacitive current density was extracted from the CV response and plotted as a function of scan rate, and the slope was used to obtain Cdl. CV curves used for comparison were recorded in the potential window of 0-0.8 V at a scan rate of 50 mV s^{-1}. Electrochemical impedance spectroscopy (EIS) measurements were carried out over a frequency range from 100000 to 0.01 Hz with an amplitude of 0.005 V.

Supplementary Figures

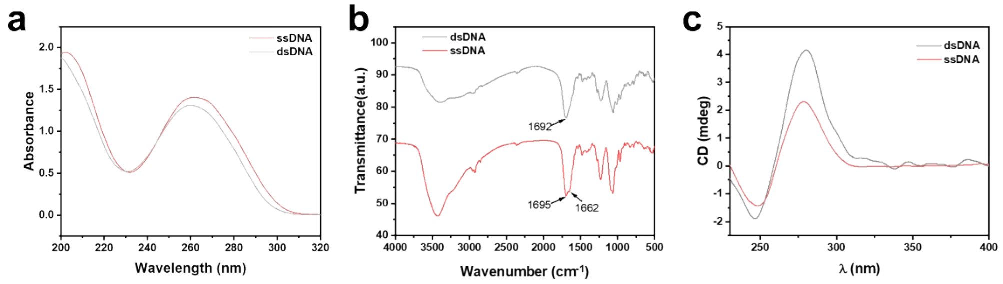

Figure S1. (a) UV-vis, (b) FT-IR, and (c) CD spectra of dsDNA and ssDNA.

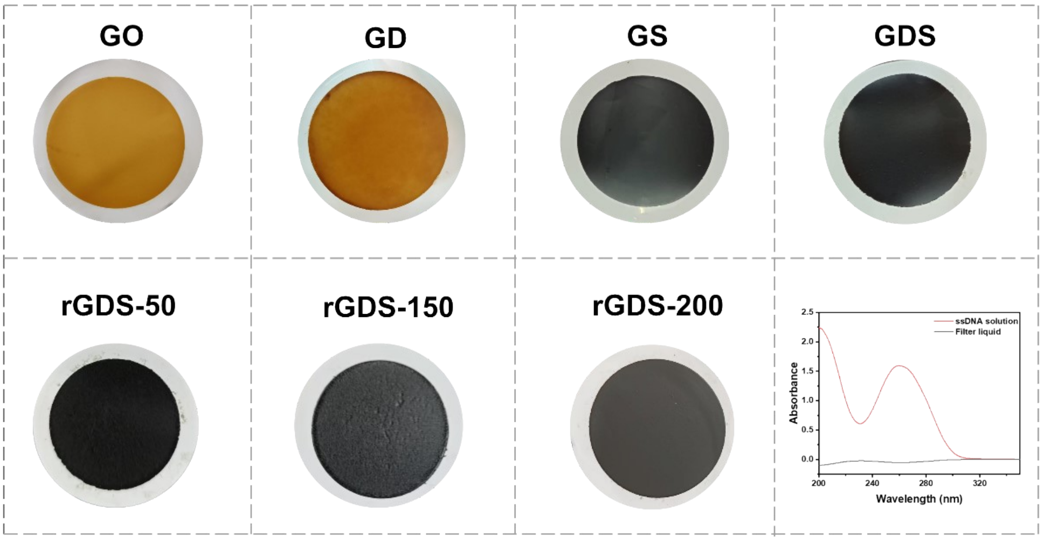

Figure S2. Photographs of membranes prepared by vacuum-assisted filtration, including GO, GD, GS, GDS, and the thermally reduced rGDS-T membranes (T = 50, 150, and 200 °C). UV-vis spectra of the ssDNA solution and the corresponding filtrate collected after membrane fabrication.

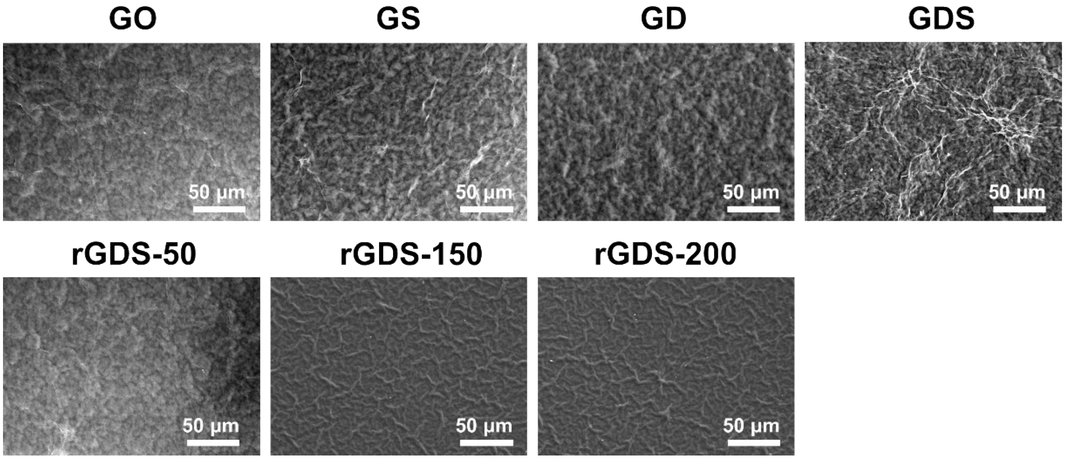

Figure S3. Surface SEM images of the as-prepared membranes, including GO, GS, GD, GDS, and the thermally reduced rGDS-T membranes (T = 50, 150, and 200 °C).

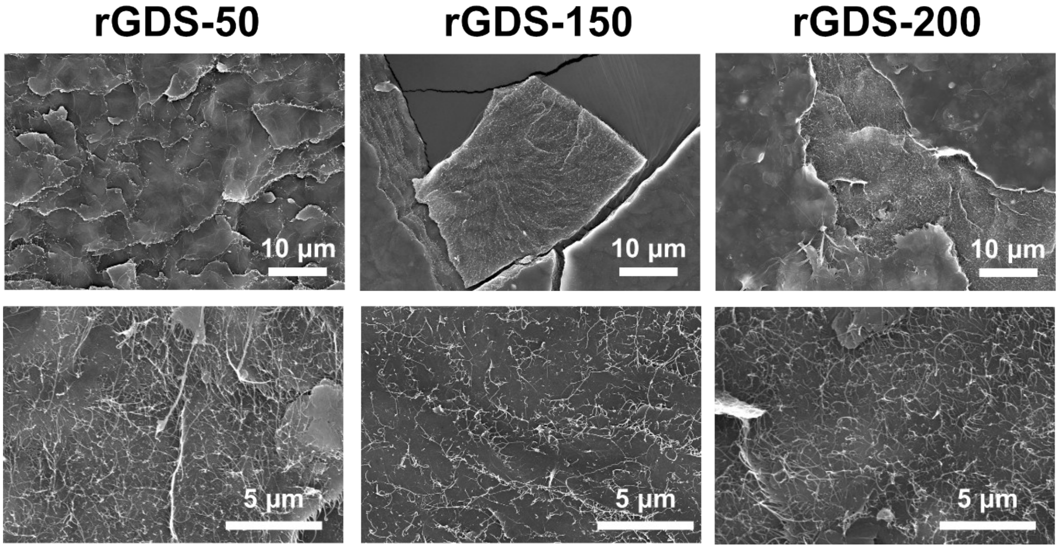

Figure S4. Surface SEM images of rGDS-T membranes (T = 50, 150, and 200 °C) after adhesive-tape peeling.

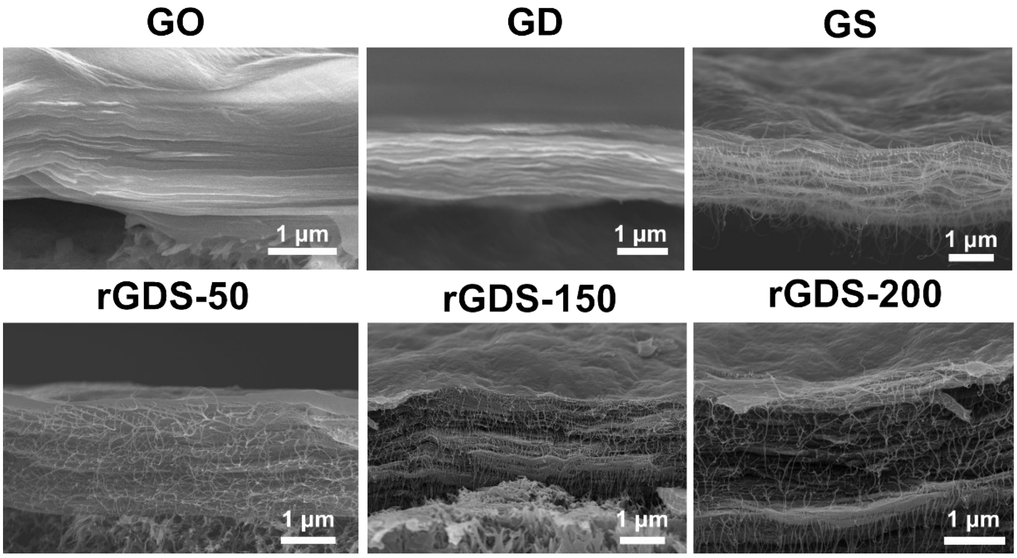

Figure S5. Cross-sectional SEM images of the GO, GD, GS, and thermally reduced rGDS-T membranes (T = 50, 150, and 200 °C).

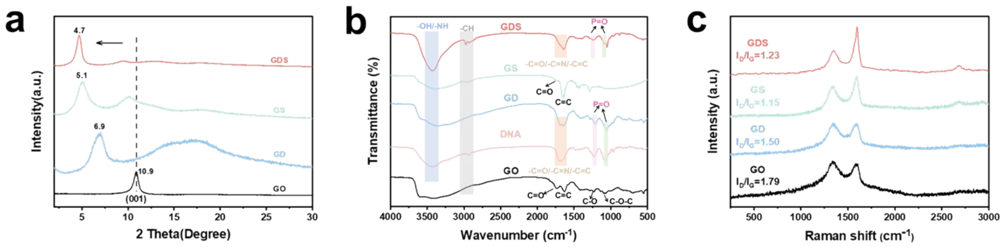

Figure S6. Structural and spectroscopic characterization of GO-based control membranes and the GDS membrane. (a) XRD patterns of GO, GD, GS, and GDS membranes. (b) FT-IR spectra of GO, ssDNA, GD, GS, and GDS. (c) Raman spectra of GO, GD, GS, and GDS.

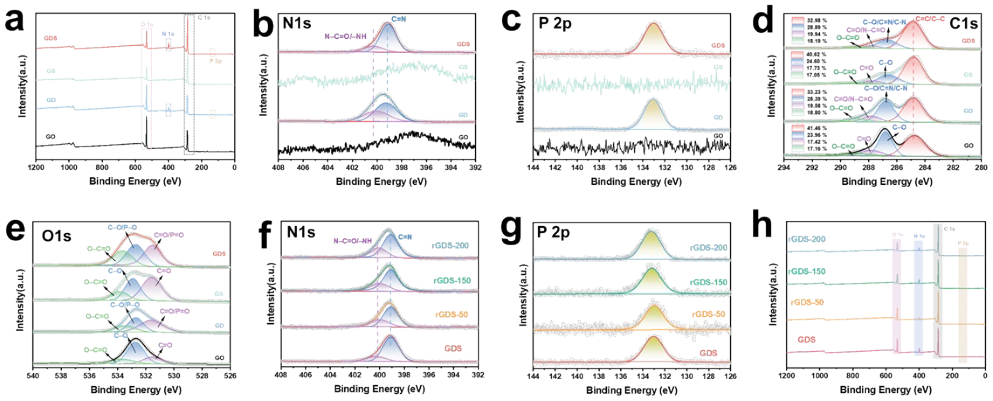

Figure S7. XPS characterization of GO-based control membranes and GDS/rGDS membranes. (a) XPS survey spectra of GO, GD, GS, and GDS. High-resolution spectra of (b) N 1s, (c) P 2p, (d) C 1s, and (e) O 1s for GO, GD, GS, and GDS. High-resolution spectra of (f) N 1s and (g) P 2p for GDS and thermally reduced rGDS membranes, together with (h) the corresponding survey spectra.

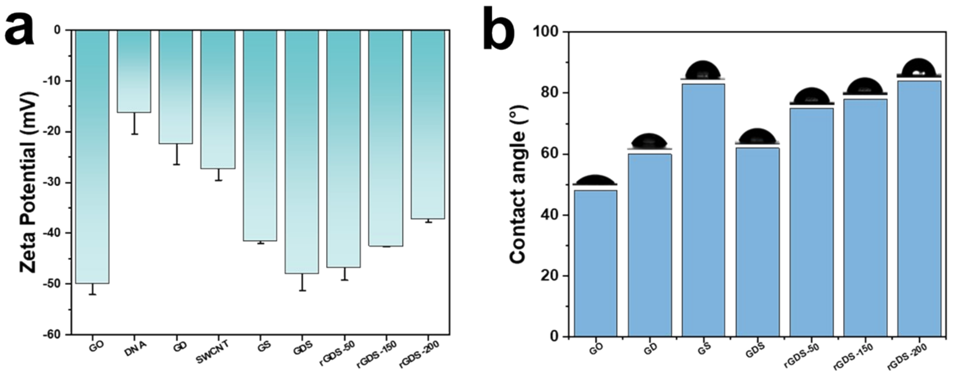

Figure S8. Interfacial properties of precursor dispersions and corresponding assembled membranes. (a) Zeta potentials of GO, ssDNA , SWCNT dispersion, and the corresponding mixed dispersions used to fabricate GD, GS, GDS, and rGDS. (b) Static water contact angles of the membranes (GO, GD, GS, GDS, and rGDS).

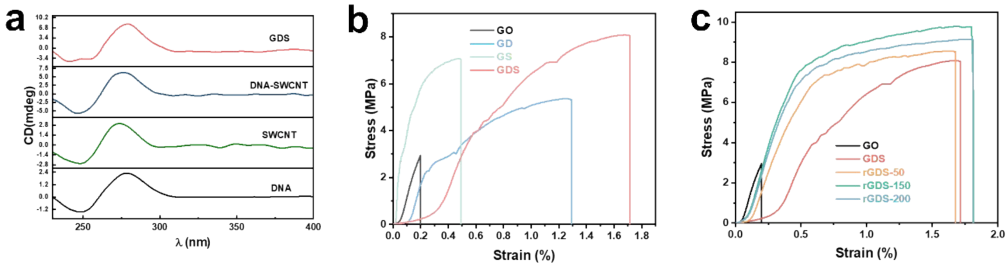

Figure S9. Chiroptical signature and tensile properties of GDS/rGDS membranes and control samples. (a) Circular dichroism (CD) spectra of ssDNA, SWCNT, the ssDNA-SWCNT complex (DNA-SWCNT), and the assembled GDS membrane. (b) Representative stress-strain curves of GO, GD, GS, and GDS membranes. (c) Representative stress-strain curves of GO, GDS, and thermally reduced rGDS-T membranes (T = 50, 150, and 200 °C).

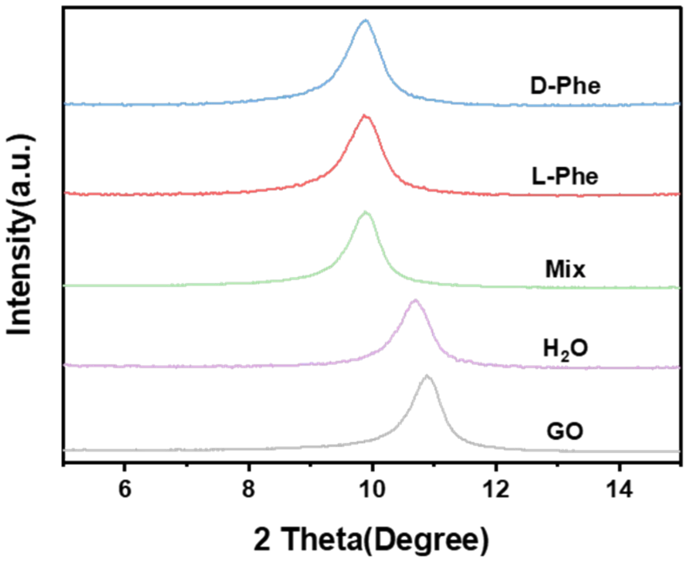

Figure S10. GO membranes were separately soaked in deionized water (H_2O), L-Phe, D-Phe, and the racemic mixture (Mix), air-dried under ambient conditions, and then subjected to XRD measurement.

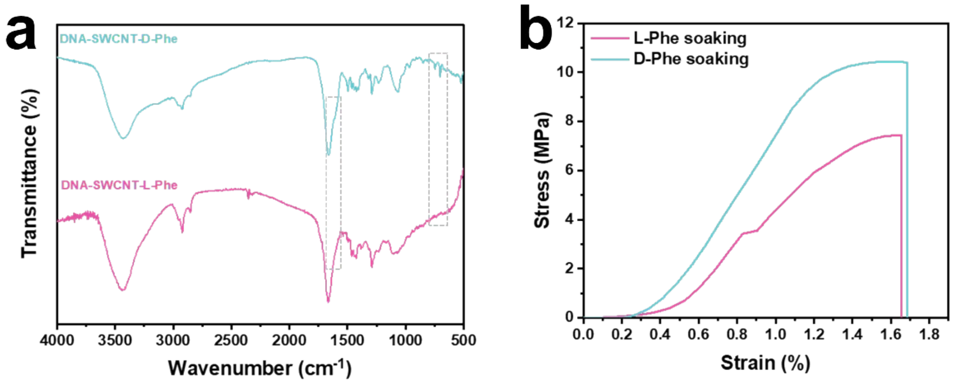

Figure S11. (a) FT-IR spectra of DNA-SWCNT after interaction with L-Phe and D-Phe solutions. (b) Representative tensile stress-strain curves of GDS membranes after soaking in L-Phe or D-Phe solutions.

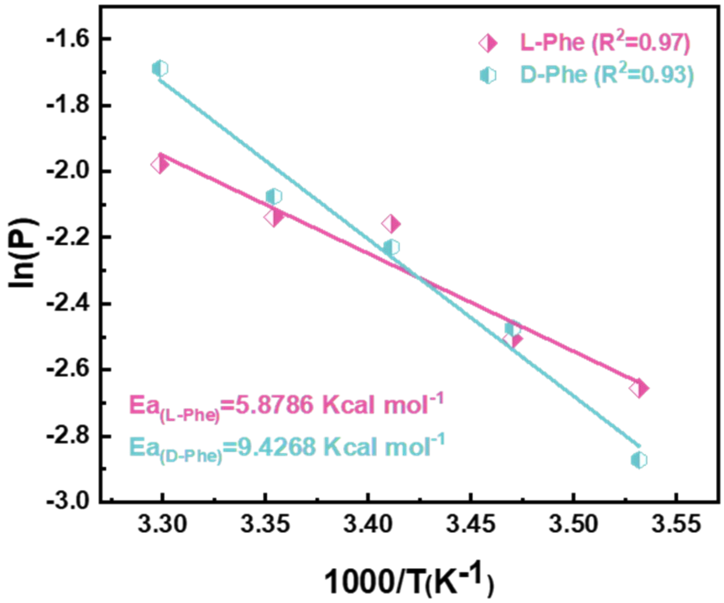

Figure S12. Arrhenius analysis of enantiomer transport through the GDS membrane.

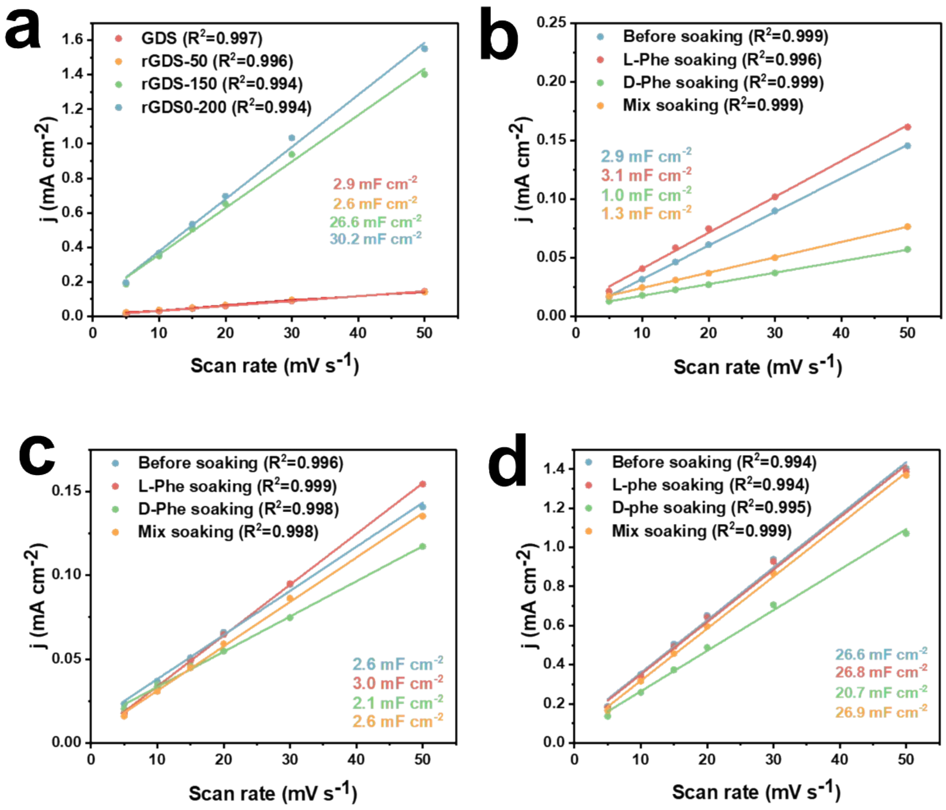

Figure S13. Double-layer capacitance (Cdl) analysis of GDS/rGDS membranes and its dependence on enantiomer soaking. (a) Determination of Cdl for GDS and thermally reduced rGDS-T membranes (T = 50, 150, and 200 °C) from the linear relationship between capacitive current density (j) and scan rate. (b-d) Cdl determination before soaking and after soaking in L-Phe, D-Phe, or racemic mixture (Mix) for (b) GDS, (c) rGDS-50, and (d) rGDS-150 membranes.

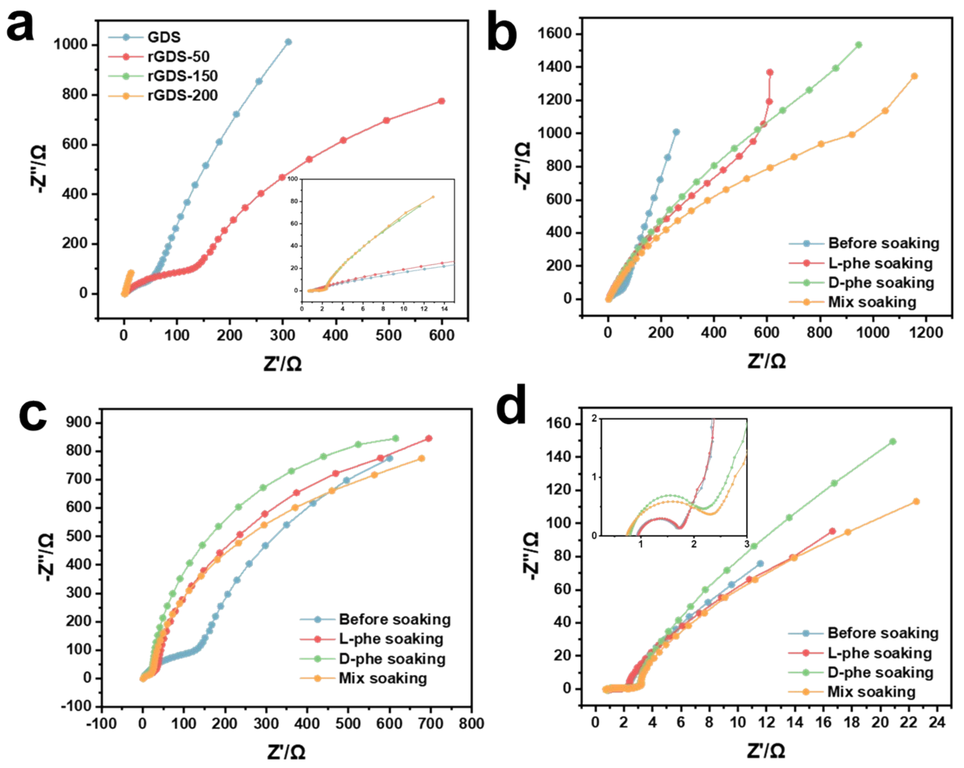

Figure S14. Electrochemical impedance spectroscopy (EIS) of GDS/rGDS membranes and their response to enantiomer soaking. (a) Nyquist plots of GDS and thermally reduced rGDS-T membranes (T = 50, 150, and 200 °C). (b-d) Nyquist plots of (b) GDS, (c) rGDS-50, and (d) rGDS-150 membranes measured before soaking and after soaking in L-Phe, D-Phe, or the racemic mixture (Mix).

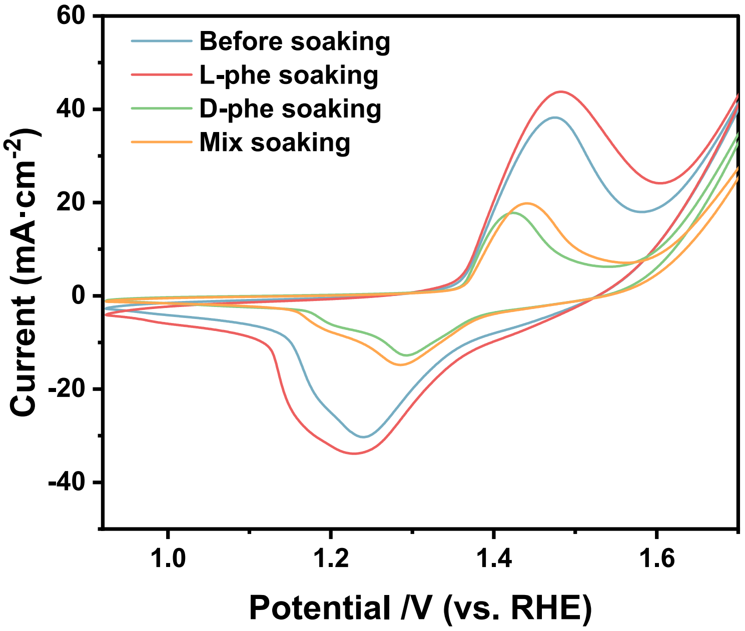

Figure S15. CV curves of rGDS-200 measured before soaking and after soaking in L-Phe, D-Phe, or the racemic mixture (Mix).

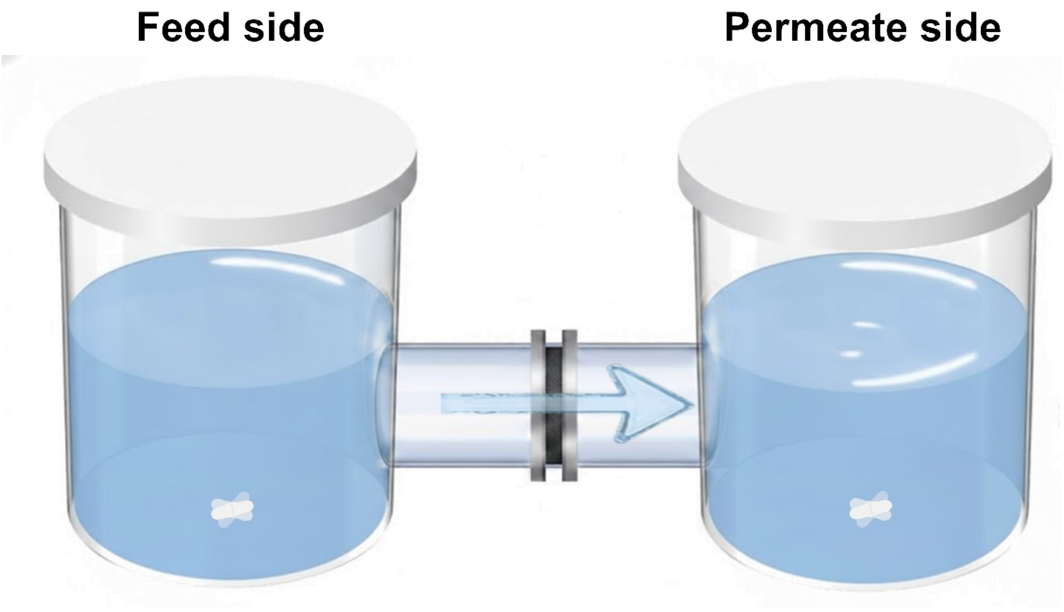

Figure S16. Schematic illustration of the custom-built permeation device for enantiomer separation. The left reservoir is the feed side containing the enantiomer-containing feed solution, and the right reservoir is the permeate (receiving) side containing the driving solution. The arrow indicates solute permeation from the feed to the receiving side.

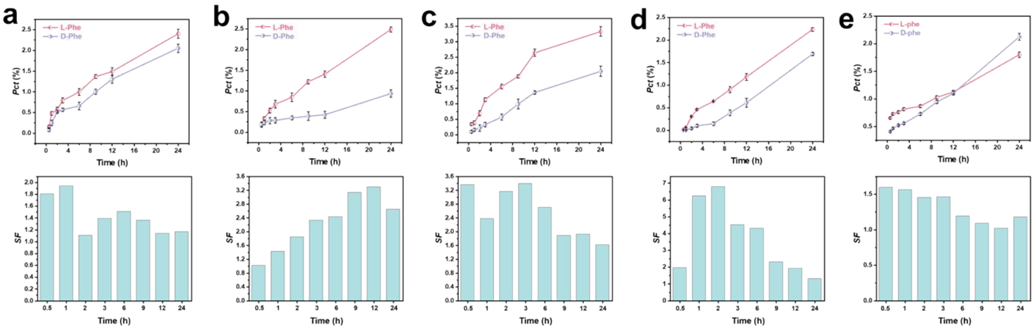

Figure S17. Time-dependent enantiomer Pct and SF for control membranes. (a-d) Permeation kinetics of L-Phe and D-Phe through (a) GO membrane, (b) GD membrane, (c) GS membrane, and (d) GDS membrane. The upper panels show the Pct as a function of time, and the lower panels present the corresponding SF calculated at each time point. (e) L-/D-Phe separation performance of the GO-dsDNA-SWCNT control membrane prepared under the same conditions as the GDS membrane.

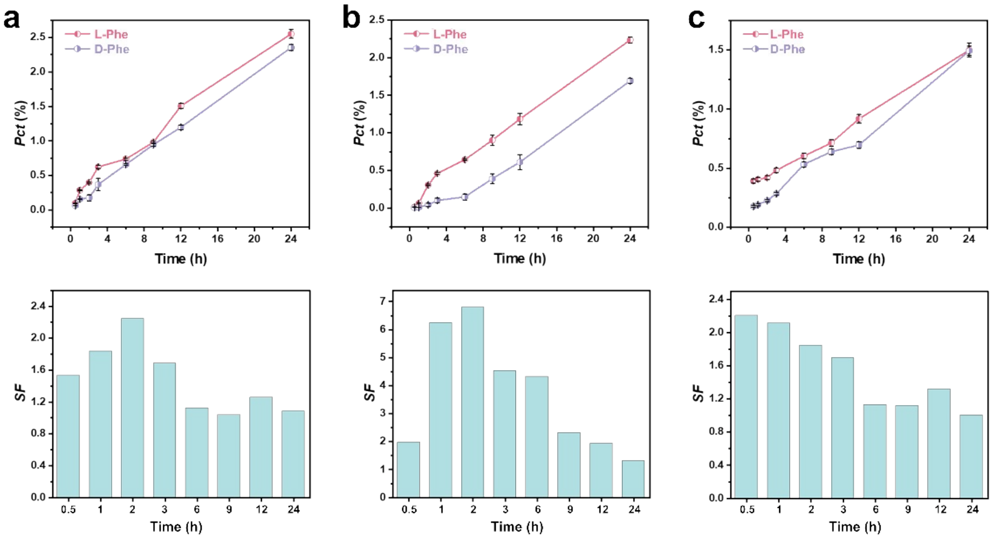

Figure S18. Effect of ssDNA loading on L/D-Phe separation by GDS membranes at a fixed SWCNT content. (a-c) Time-dependent Pct of L-Phe and D-Phe (top panels) and the corresponding SF (bottom panels) for GDS membranes prepared with different ssDNA loadings: (a) 5 mg, (b) 10 mg, and (c) 20 mg ssDNA.

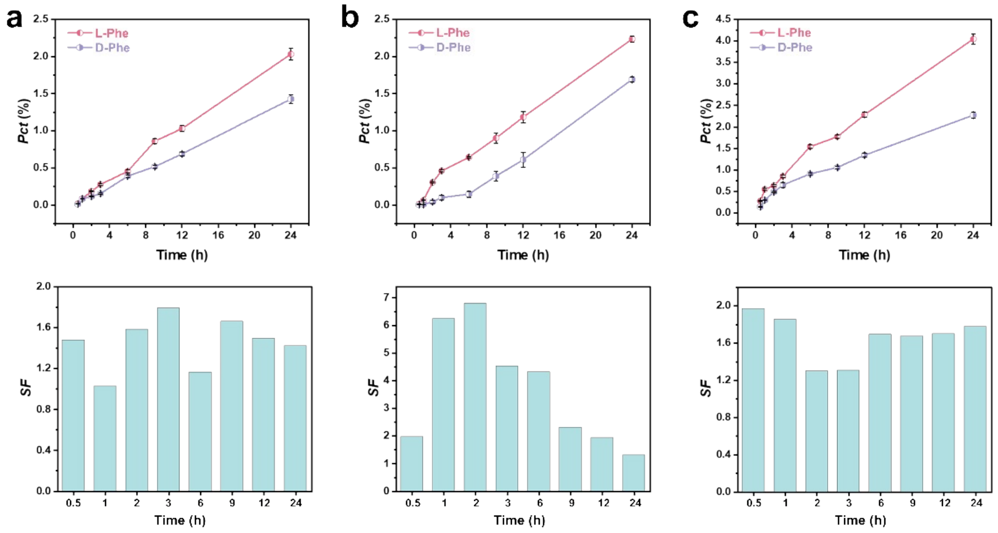

Figure S19. Effect of SWCNT loading on L/D-Phe separation by GDS membranes at a fixed ssDNA content. (a-c) Time-dependent Pct of L-Phe and D-Phe (top panels) and the corresponding SF (bottom panels) for GDS membranes prepared with different SWCNT loadings: (a) 0.5 mg, (b) 1.0 mg, and (c) 2.0 mg SWCNT.

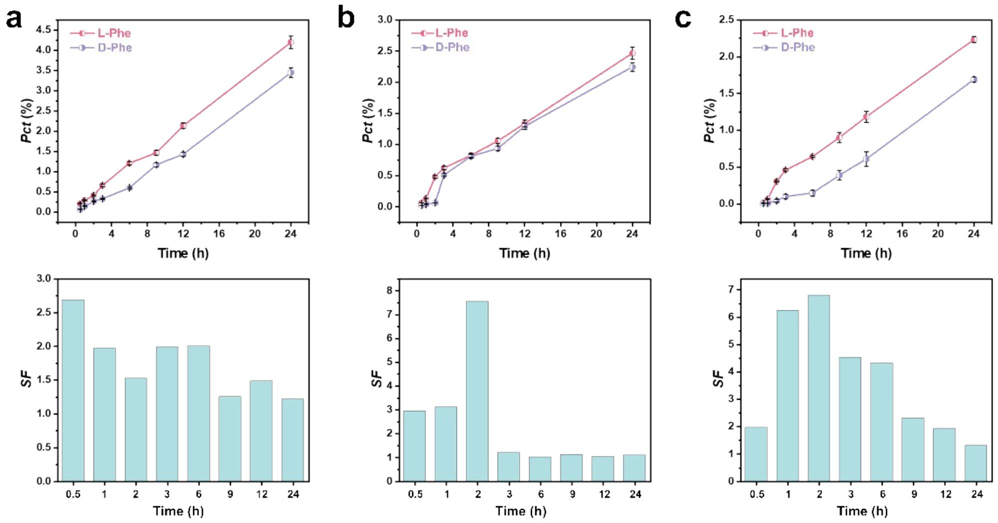

Figure S20. Effect of the driving-solution pH on L/D-Phe separation by the GDS membrane. (a-c) Time-dependent Pct of L-Phe and D-Phe through the GDS membrane (top panels) and the corresponding SF (bottom panels) measured with driving (receiving-side) solutions at different pH values: (a) pH 2, (b) pH 4, and (c) pH 6.

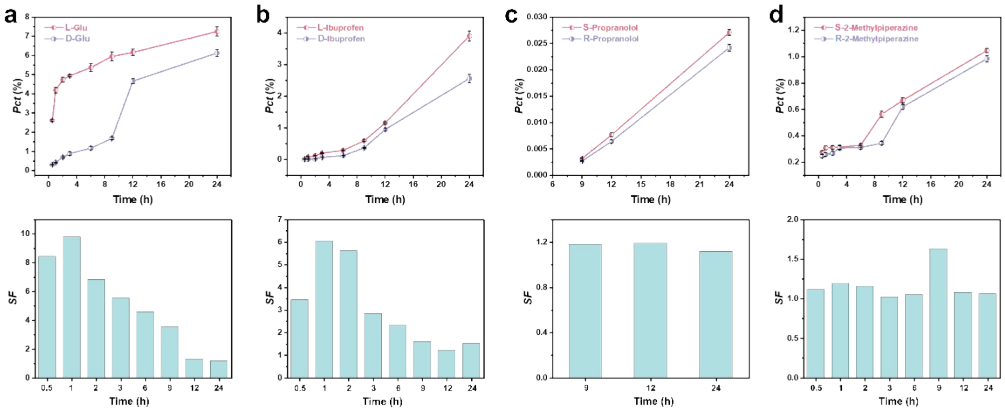

Figure S21. Separation of representative chiral amino acids and pharmaceuticals by the GDS membrane. (a-d) Time-dependent Pct (top panels) and the corresponding SF (bottom panels) for enantiomer pairs: (a) L/D-Glu, (b) R/S-Ibuprofen, (c) R/S-Propranolol, and (d) R/S-2-methylpiperazine.

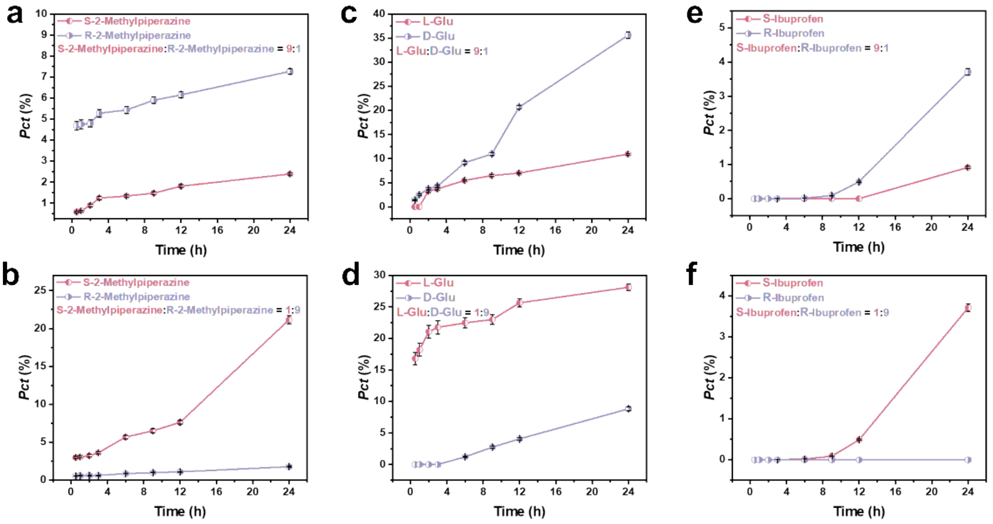

Figure S22. Permeation kinetics of enantiomer-enriched mixtures through the GDS membrane. (a,b) Time-dependent Pct of S/R-2-methylpiperazine at different enantiomeric ratios: S:R = 9:1 (a) and 1:9 (b). (c,d) Pct-t curves of L/D-Glu at L:D = 9:1 (c) and 1:9 (d). (e,f) Pct-t curves of S/R-Ibuprofen at S:R = 9:1 (e) and 1:9 (f).

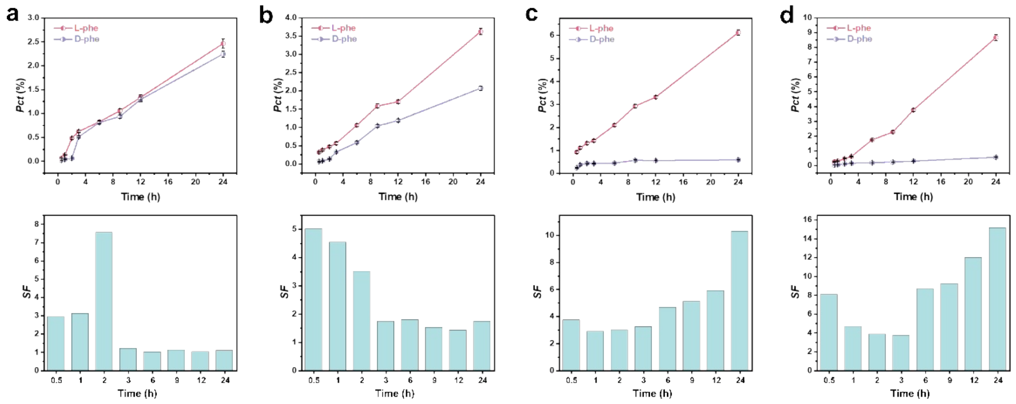

Figure S23. Comparative permeation kinetics and separation factors of L/D-phenylalanine across GDS and rGDS-T membranes. (a-d) Time-dependent Pct (top panels) of L-Phe and D-Phe, together with the corresponding SF (bottom panels), for (a) GDS, (b) rGDS-50, (c) rGDS-150, and (d) rGDS-200 membranes.

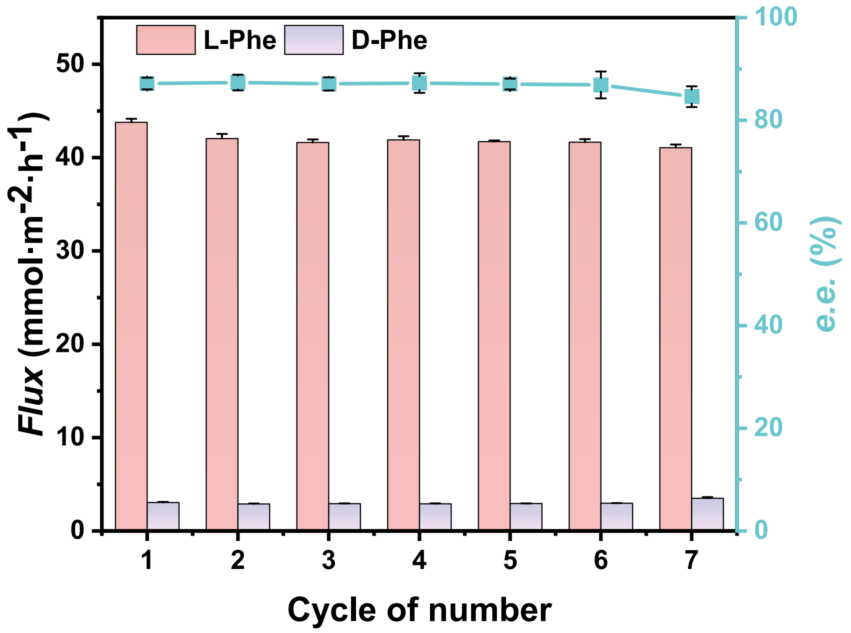

Figure S24. Cycling stability of rGDS-200 for enantioselective phenylalanine permeation.

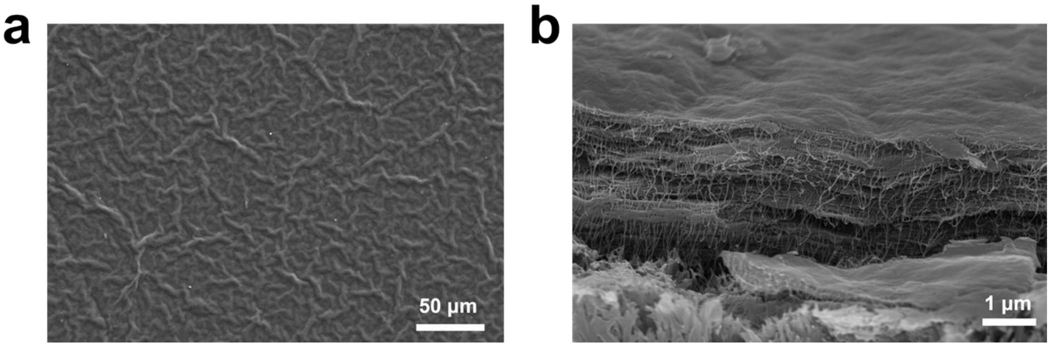

Figure S25. SEM characterization of the rGDS-200 membrane after 168 h cycling operation. (a) Surface SEM image and (b) cross-sectional SEM image of the rGDS-200 membrane after long-term enantioselective permeation.

Supplementary Tables

Table S1. Comparison of tensile mechanical performance among representative membranes

Strain enhancement	Tensile strength enhancement	Modified membrane	Reference (baseline)	Ref.
2.54	1.55	UHMWPE/MXene	UHMWPE	4
2.83	1.18	PTMG-HDI-BHDS polymer electrolyte	PTMG–HDI	5
6.26	0.92	a-EG/PEDOT:PSS (with $NaClO_4$ treatment)	a-EG/PEDOT:PSS without $NaClO_4$ treatment	6
2.58	1.05	PP/PE/ML-MOF (5:5)	PP/PE	7
4.56	2.74	S-SBM	S-MXene	8
2.65	3.60	PDG-UPy-IV	GO	9
2.19	2.37	GO/PGA/Ca^{2+}	GO	10
2.43	1.96	CCC-GO	GO	11
6.42	7.15	rGO-BP-AD	GO	12
2.73	2.28	GO-BP	GO	12
1.15	1.40	PBO/MXene	PBO	13

Notes: Strain enhancement = $\varepsilon_{\text{modified}}/\varepsilon_{\text{baseline}}$

Tensile strength enhancement = $\sigma_{\text{modified}}/\sigma_{\text{baseline}}$

Table S2. Fitted EIS parameters of rGDS-200 membranes.

**Condition**	Rs (Ω)	Rct (Ω)	Wo-R (Ω)	Wo-T (s)	Wo-P
Before	0.70	0.86	1.44	0.20	0.46
L-Phe	0.95	0.81	1.32	0.19	0.46
D-Phe	0.98	3.90	1.45	0.12	0.46
Mix	1.01	0.87	1.72	0.24	0.46

Table S3. Comparison of enantioselective membrane separation performance in representative porous-framework membranes

**Chiral separation membrane**	**Feed concentration (M)**	**Separation target**	**e.e. (%)**	**Time to reach listed e.e., t ee (h)**	**Flux (mmol • m^{-2} • h^{-1})**	**Ref.**
TAH-COF	0.002	threonine	94.3	2	1.42	14
TpCM-COF	0.005	Phenylalanine	99.4	2	0.03	15
CC3-HPABP-POC	0.10	2-Phenylpropionic acid	100	0.25	2.93	16
CC3-*R*/PA-POC	0.01	limonene	95.2	5	0.73	17
MOF-808	0.001	naproxen	95.0	NR	1.8×10^{-3}	18
MIL-53-NH-L-His -MOF	0.01	1-Phenylethanol	100	2	0.25	19
l-His-ZIF-8-MOF	0.008	1-Phenylethanol	76	2	5.112	20
Mb-COF	0.01	PRF	96.4	NR	0.367	21
rGDS-200	0.018	Phenylalanine	87.2	24	42.7	This work

Notes: NR indicates that the permeation/sampling time corresponding to the listed e.e. was not reported.

References

1 F. Yuan, Q. Gao, Z. Lv, Y. Zhang, X. Liu, J. Peng and Z. Li, *Nano Lett.*, 2024, **24**, 14346–14354.

2 T. Liu, X. Zhang, J. Liang, W. Liang, W. Qi, L. Tian, L. Qian, Z. Li and X. Chen, *Nano Lett.*, 2023, **23**, 9641–9650.

3 X. Li, Q. Mu, R. Li and Y. Ji, *Anal. Chem.*, 2025, **97**, 21598–21607.

4 X. Liu, W. Zhang, X. Zhang, Z. Zhou, C. Wang, Y. Pan, B. Hu, C. Liu, C. Pan and C. Shen, *Nat. Commun.*, 2024, **15**, 3076.

5 F. Pei, L. Wu, Y. Zhang, Y. Liao, Q. Kang, Y. Han, H. Zhang, Y. Shen, H. Xu, Z. Li and Y. Huang, *Nat. Commun.*, 2024, **15**, 351.

6 H. He, R. Chen, S. Yue, S. Yu, J. Wei and J. Ouyang, *Sci. Adv.*, 2022, **8**, eabq8160.

7 T. Kuang, H. Guo, W. Guo, W. Liu, W. Li, M. R. Saeb, M. Vatankhah-Varnosfaderani and S. S. Sheiko, *Adv. Sci.*, 2024, **11**, 2407593.

8 S. Wan, Y. Chen, C. Huang, Z. Huang, C. Liang, X. Deng and Q. Cheng, *Nature*, 2024, **634**, 1103–1110.

9 Y. Wang, T. Li, P. Ma, S. Zhang, H. Zhang, M. Du, Y. Xie, M. Chen, W. Dong and W. Ming, *ACS Nano*, 2018, **12**, 6228–6235.

10 K. Liang, E. M. Spiesz, D. T. Schmieden, A.-W. Xu, A. S. Meyer and M.-E. Aubin-Tam, *ACS Nano*, 2020, **14**, 14731–14739.

11 J. Zhong, W. Sun, Q. Wei, X. Qian, H.-M. Cheng and W. Ren, *Nat. Commun.*, 2018, **9**, 3484.

12 T. Zhou, H. Ni, Y. Wang, C. Wu, H. Zhang, J. Zhang, A. P. Tomsia, L. Jiang and Q. Cheng, *Proc. Natl. Acad. Sci. U.S.A.*, 2020, **117**, 8727–8735.

13 Y. Liu, W. Zou, N. Zhao and J. Xu, *Nat. Commun.*, 2023, **14**, 5342.

14 T. Xu, L. Cao, S. An, X. Liu, Z. Li and Z. Lai, *Nat. Commun.*, 2025, **16**, 7803.

15 N. Manoranjan, W. Fang, Y. Zhu and J. Jin, *Angew. Chem. Int. Ed.*, 2025, **64**, e202417088.

16 Y. Li, Z. Liu, D. Zhu, Y. Xia, B. Wu, T. Xu, X. Li and T. Xu, *Nat. Commun.*, 2025, **16**, 11699.

17 F. Wang, K. He, R. Wang, H. Ma, P. J. Marriott, M. R. Hill, G. P. Simon, M. M. B. Holl and H. Wang, *Adv. Mater.*, 2024, **36**, 2400709.

18 T. Chen, H. Li, X. Shi, J. Imbrogno and D. Zhao, *J. Am. Chem. Soc.*, 2024, **146**, 14433–14438.

19 Y. Lu, H. Zhang, J. Y. Chan, R. Ou, H. Zhu, M. Forsyth, E. M. Marijanovic, C. M. Doherty, P. J. Marriott, M. M. B. Holl and H. Wang, *Angew. Chem. Int. Ed.*, 2019, **58**, 16928–16935.

20 J. Y. Chan, H. Zhang, Y. Nolvachai, Y. Hu, H. Zhu, M. Forsyth, Q. Gu, D. E. Hoke, X. Zhang, P. J. Marriot and H. Wang, *Angew. Chem. Int. Ed.*, 2018, **57**, 17130–17134.

21 L. Yang, Y. Feng and Y. Sun, *J. Membr. Sci.*, 2026, **741**, 125044.
